# Measuring endemicity and burden of leprosy across countries and regions: A systematic review and Delphi survey

**DOI:** 10.1371/journal.pntd.0009769

**Published:** 2021-09-20

**Authors:** Dorcas O. Ogunsumi, Vivek Lal, Karl Philipp Puchner, Wim van Brakel, Eva-Maria Schwienhorst-Stich, Christa Kasang, Joseph Chukwu, Saskia Kreibich, Sandra Parisi, Jan Hendrik Richardus, David J. Blok

**Affiliations:** 1 Department of Public Health, Erasmus MC, University Medical Center Rotterdam, Rotterdam, Netherlands; 2 Sasakawa-India Leprosy Foundation, New Delhi, India; 3 German Leprosy and Tuberculosis Relief Association, Würzburg, Germany; 4 Medical Faculty/Master’s Programme Global Health and Disaster Medicine, University of Athens, Greece; 5 NLR International, Amsterdam, Netherlands; 6 Faculty of Medicine, University of Würzburg, Würzburg, Germany; 7 Department for General Practice, Universitätsklinikum Würzburg, Würzburg, Germany; Federal University of Bahia - Institute of Collective Health, BRAZIL

## Abstract

**Background:**

Leprosy is a chronic infectious disease caused by *Mycobacterium leprae*, the annual new case detection in 2019 was 202,189 globally. Measuring endemicity levels and burden in leprosy lacks a uniform approach. As a result, the assessment of leprosy endemicity or burden are not comparable over time and across countries and regions. This can make program planning and evaluation difficult. This study aims to identify relevant metrics and methods for measuring and classifying leprosy endemicity and burden at (sub)national level.

**Methods:**

We used a mixed-method approach combining findings from a systematic literature review and a Delphi survey. The literature search was conducted in seven databases, searching for endemicity, burden and leprosy. We reviewed the available evidence on the usage of indicators, classification levels, and scoring methods to measure and classify endemicity and burden. A two round Delphi survey was conducted to ask experts to rank and weigh indicators, classification levels, and scoring methods.

**Results:**

The literature review showed variation of indicators, levels, and cut-off values to measure leprosy endemicity and/or burden. The most used indicators for endemicity include new case detection rate (NCDR), new cases among children and new cases with grade 2 disability. For burden these include NCDR, MB cases, and prevalence. The classification levels ‘high’ and ‘low’ were most important. It was considered most relevant to use separate scoring methods for endemicity and burden. The scores would be derived by use of multiple indicators.

**Conclusion:**

There is great variation in the existing method for measuring endemicity and burden across countries and regions. Our findings contribute to establishing a standardized uniform approach to measure and classify leprosy endemicity and burden at (sub)national level, which would allow effective communication and planning of intervention strategies.

## Author summary

Leprosy is a chronic infectious disease caused by *Mycobacterium leprae*. If left untreated, it could lead to deformity and resultant disabilities. The number of new cases has remained stagnant for the past decade. Additional interventions are required tailored to the level of endemicity and burden of an area. Currently, measuring and classifying endemicity and burden in leprosy lacks a standardized uniform approach, making program planning and evaluation difficult. We did a systematic literature search and Delphi survey, a method to elicit (group) opinion from a panel of experts, to take an inventory of indicators, classification levels and cut-offs that were used to measure and classify endemicity and burden of leprosy. This study highlights the variation in usage of indicators, levels, and cut-off values to determine the endemicity and burden of leprosy across regions and countries. There was agreement that endemicity and burden should be measured separately using multiple indicators, and that the classification levels ‘high’ and ‘low’ were most important. Our findings contribute to establishing a standardized uniform approach to measure and classify leprosy endemicity and burden at (sub)national level, which would allow effective communication and planning of intervention strategies.

## Introduction

Leprosy is a chronic, infectious disease caused by the bacterium *Mycobacterium leprae*, which primarily affects the skin and peripheral nerves. [1] The incubation period is long and variable, it is assumed to be five years on the average but could be up to 20 years before occurrence of symptoms. [2] If left untreated, it could lead to deformity and resultant disabilities. [3]

Over the past decade, global leprosy control and elimination strategies have been based on specific indicators. The WHO target for global elimination of leprosy as a public health problem by the year 2000 was described in terms of the reduction of prevalence to a level below one case per 10 000 population. [4] The same target was later applied to national level to ensure elimination goal was attained by the end of 2005. In recognition of importance of timely detection of cases, the target was later changed to reduction of the rate of new cases with grade 2 disability (G2D) by at least 35% by the end of 2015 (‘Enhanced global strategy to further reduce the disease burden due to leprosy 2011–2015’).[5] The targets of London Declaration on Neglected Tropical disease (NTDs) for leprosy included global interruption of transmission and reduction of G2D in newly detected cases to less than one per million.[6] The Global Leprosy Strategy 2016–2020 aimed at achieving zero disability among children, reduction in global G2D to less than one per million and repealing of discriminatory laws.[7] A set of 13 core programmatic indicators was defined, viz. annual new case detection, annual new case detection rate, prevalence, prevalence rate, proportion of G2D, paediatric cases, MB cases, females and foreign-born cases among new cases, number of relapses reported in a year, treatment completion/cure rate of MB and PB cases and percentage of contacts screened among the household contacts registered. The indicators were related to case detection and ability to detect early, ongoing transmission and clinical presentations.

The global annual number of new cases detected in 2019 was 202,189. [8] There is optimism that scaling up of campaigns for early case detection in several countries and initiatives to cover populations at risk with chemoprophylaxis will accelerate attainment of a world without leprosy. [9] The new global leprosy strategy 2021–2030 will include prevention of leprosy by preventive chemotherapy of contacts and other high-risk groups. Many of these interventions require an accurate definition of endemicity. Leprosy is unevenly distributed globally, with India, Brazil, and Indonesia accounting for 80% of the total number of new cases. Furthermore, within countries, it is focalised, with pockets of high endemicity existing often at sub-district level, despite relatively low country estimates. To optimise the outcome of new/additional interventions, there is the need to appropriately channel effective and feasible strategies to the respective high and low endemic situations.

Currently, measuring and classifying endemicity and burden in leprosy lack a uniform approach. In some areas the metrics used may even change over the years, making program evaluation difficult. As a result, the assessment of leprosy endemicity or disease burden is not comparable over time and across countries and regions. Establishing a uniform approach to measure and classify of leprosy endemicity and burden at a subnational level is needed to understand the leprosy situation as accurately as possible so that leprosy control activities can be tailored accordingly. A uniform approach is important for allowing effective communication, creating a monitoring framework, and ultimately assessing elimination of leprosy. Moreover, it can assist in policy decisions regarding intervention strategies and resource allocation within in a country.

This study aimed 1) to systematically review the indicators, the classification levels and scoring methods used to measure and classify endemicity and/or burden at subnational level using routine programmatic data, and 2) to obtain a balanced account of their (relative) importance for measuring endemicity and burden of leprosy through a Delphi expert study.

## Methods

### Systematic review

A systematic literature search was conducted in January 31^st^, 2018 (and updated in July 13^th^, 2020) using *Embase*, *Medline Ovid*, *Web of science*, *Cochrane central*, *Lilacs*, *Scielo and Google Scholar*. The following search terms were used: ‘leprosy’, ‘Hansen’s disease’, ‘Mycobacterium leprae’, ‘endemicity’, ‘hyperendemic’, ‘burden’. The search strategy was adapted for each database. In addition, records were included via cross-references from published papers and reports. The complete search strategy can be found in S1 Table.

All studies that met the inclusion criteria were included with no restriction to study designs except for systematic reviews from 1990 onwards retrieved by this search. There were no restrictions on the language of the articles. Each article needed to meet the following inclusion criteria: 1) it described endemicity and/or burden of leprosy using leprosy epidemiologic indicators, and/or 2) it classified levels of endemicity and/or burden based on indicators cut-off values. We excluded studies on: 1) pathogenesis and histopathological lesion of leprosy, (ii) serological and molecular evaluation, (iii) symptoms and clinical manifestations (case reports), (iv) diagnostics and immunological reactions, (vi) drug/treatment regimens, and (vii) systematic reviews. The selection of studies was carried out by two independent investigators. Disagreements between investigators were resolved by consensus. We registered our systematic review protocol at PROSPERO (Registration number: CRD42019104933). [10]

We extracted and assessed the following data for each article: first author and year of publication, country, study setting, the indicators used to measure endemicity and/or burden, the classification levels used, and the scoring method used to classify endemicity and/or burden. The scoring method was further assessed based on: 1) use of any existing published scoring method, 2) indicator cut-off values to define endemicity and/or burden levels, and 3) whether a single, multiple or composite indicator scoring method was used. Data obtained from separate studies using the same indicators and scoring method were combined. The data was summarized in tables and frequency plots.

### Delphi survey

A two round survey was conducted to collect and prioritise expert’s opinions about methods and indicators for classifying endemicity and burden of leprosy. Sampling of experts was purposeful, and this included professionals with a proven track record in the field of leprosy, including academic researchers (known experts in the field of leprosy), clinician national program managers/NGO staff, policy makers, and independent consultants. The surveys were sent out by email through *Lime survey*, an online survey tool (limesurvey.org). Experts were provided with a link and unique access code, which allowed blinding of the moderators when processing responses. In total, forty experts were approached. The first round was sent out in December 2018, and the second round in June 2019. The study did not need IRB approval since it did not concern medical (patient) research nor affected participants. The responses were analysed anonymously.

The first-round survey contained open questions regarding concepts of endemicity and burden of leprosy, indicators to measure endemicity and burden, levels for classification of endemicity and burden, and indicator scoring methods. It also covered general items including occupation, country at work, and years of experience in the field of leprosy (See S1 File).

For second-round survey, results of systematic review and first-round survey were used. Experts were asked to rank indicators (rank 1 to 10), and to score different usage of indicator values (five-point scale), the importance of indicator cut-off values (five-point scale), the relevance of proposed classification levels and scoring methods (three-point scale) (See S2 File).

Delphi questionnaire items were analysed individually. For the first-round, a simple content analysis was conducted, i.e., the essential details from each response were noted and then the frequencies of common expressions were recorded. The answers most frequently suggested were then used to form definitions/concepts for the second-round survey.

For the second-round, we identified the most important indicators based on two criteria: 1) mean ranking scores (rank 1 to 5 were given 5 to 1 point, respectively), and 2) the percentage of respondents that ranked a particular indicator in the Top-3. Statements about the usage of indicator values and indicator cut-offs were evaluated based on: 1) mean scores (strongly agree = 4, agree = 3, neutral = 2, disagree = 1, strongly disagree = 0), and 2) the percentage that agreed with the statement. The relevance of various classification levels and methods of indicator scoring were assessed based on: 1) mean scores (highly relevant = 2, relevant = 1, not relevant = 0), and the percentage that scores at least “relevant” for a particular item.

## Results

**Fig 1:**
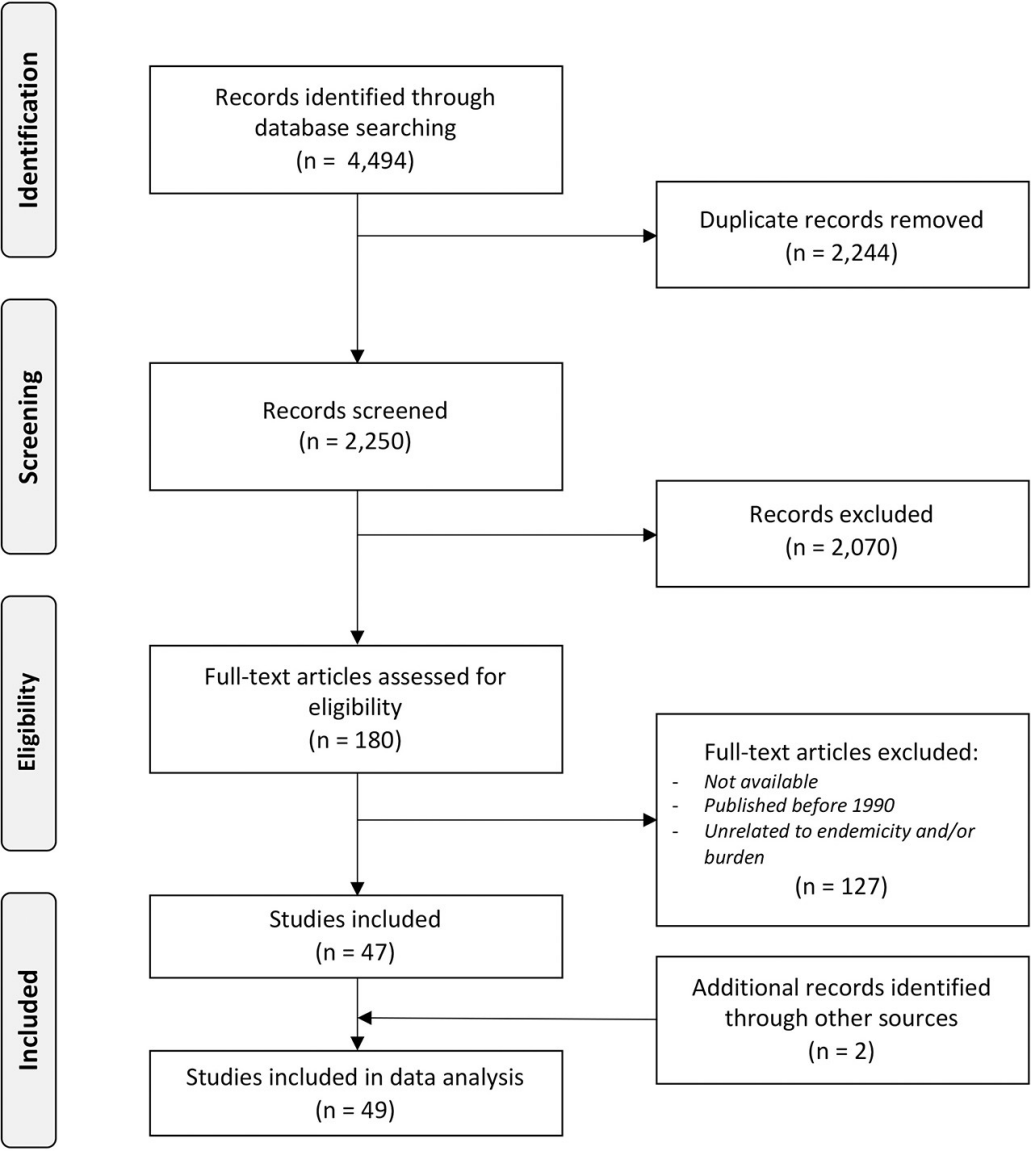
PRISMA Flow Diagram of systematic literature review.

The systematic literature search in all databases yielded 4,594 articles, of which 2,244 were duplicates. The remaining 2,250 articles were screened by title and abstract, of which 180 articles were eligible. After the full-text analysis, we included 47 articles. 23 from Brazil [11–33], 6 from India [34–39], 3 from China [40–42], 2 from Bangladesh [2,43] and Comoros [44,45]. One from Cambodia [46], Sri-Lanka [47], Cameroon [48], Venezuela [49], Bangladesh and Thailand [50], Indonesia [51], Argentina [52], and Uganda [53]. Three publications focussed on the global leprosy burden. [54–56] In addition, two reports (grey literature) were identified through other sources (Fig 1). [57,58]

The response rate of the Delphi survey was 65% (26 out of 40) in the first round, and 69% (18 out of 26) in the second round. Participants of the Delphi survey included researchers (n = 6; 23%), clinician (n = 7; 27%), NGO staff (n = 7; 27%), policy makers (n = 1; 4%), and others such as consultants (n = 5; 19%). Twenty seven percent of the participants worked between 1 and 14 years in the field of leprosy, 50% between 15 and 30 years, and 23% more than 30 years. Together they worked in more than 40 endemic countries. Detailed results of round 1 and 2 can be found in S1 and S2 Files.

### Endemicity and burden of leprosy

In the systematic review, endemicity and burden of leprosy were described by the same indicators, indicating that there was no clear/sharp distinction between both concepts. After one round of Delphi survey, a great majority agreed that endemicity and burden of leprosy were two different concepts (96%) (See S1 File). Endemicity was associated with indicators reflecting the number of cases in an area, while burden of leprosy has a broader interpretation that primarily focused on the number of people living with disability and societal consequences, such as stigma. Besides, the concept of endemicity and burden, other concepts were suggested, including hidden endemicity, economic burden or cost of leprosy, and quality of leprosy services.

### Existing methods for measuring and classifying endemicity and burden of leprosy

Two existing classification methods for endemicity were identified in the systematic review. The first method is the Leprosy Burden Scale (LBS) developed by the WHO Regional Office for Africa. This method provided cut-off scores for nine indicators into three endemicity levels: high, medium, and low (See Table 1). The endemicity level of each indicator was then combined into a composite score reflecting the overall endemicity in an area. The cut-off value for each indicator ranges from 0 = low endemicity level, 1 = medium level, and 2 = high level. Total sum of these values and endemicity level for the nine indicators is used to determine the overall endemicity level of an area. This method was used by one study from Cameroon. [48]

**Table 1:** Existing frameworks for classifying endemicity and burden using pre-defined levels, indicators, and cut-offs.

	WHO-AFRO Leprosy burden scale			Brazilian method				
Indicators	High	Medium	Low	Hyper-endemic	Very High	High	Medium	Low
NCDR per 100,000 population	>20	10–20	<10	>40.00	20.00–39.99	10.00–19.99	2.00–9.99	<2.00
NCDR in children 100,000 population	>20	10–20	<10	>10.00	5.00–9.99	2.50–4.99	0.50–2.49	<0.50
Prevalence rate per 10,000	>2	1–2	<1	20.0	10.0–19.99	5.0–9.9	1.0–4.9	<1.0
Proportion G2D among new cases per 100,000 population	>20	10–20	<10	NS	NS	≥10	5–9.9	<5
Proportion MB among new cases	<50	50–75	76–100	*Indicator not included*				
Proportion of females among new	<40	>60	40–60	*Indicator not included*				
Proportion of PB / MB cases cured in the year with degree of incapacity II*	*Indicator not included*			-	-	≥10	5–9.9	<5
Proportion of G2D cases cured at time of discharge	*Indicator not included*			-	-	≥10	5–9.9	<5
Detection (new cases) in regions	>100	21–100	0–20	*Indicator not included*				
Detection (new cases) in health district	>20	11–20	0–10	*Indicator not included*				
Prevalence/ detection (p/d)	>2	1–2	<1	*Indicator not included*				
G2D rate per 100,000 population	>1	0.5–1	<0.5	*Indicator not included*				

NCDR = new case detection rate; G2D = grade-2 disability; MB = multibacillary leprosy; PB = paucibacillary leprosy

The second classification method was published by the Brazilian Ministry of Health (Brazilian method) in 2002, and updated in 2009. [58] The updated classification method proposed six epidemiological indicators to classify endemicity, and in addition five operational indicators to monitor the quality of actions and services (See Table 1). Each epidemiologic indicator had cut-off values for five endemicity levels: hyper-endemic, very high, high, medium, and low. The endemicity level of an area was based on one or more of the proposed epidemiological indicators, and endemicity level was determined separately per indicator. In total, 22 studies from Brazil followed the Brazilian guidelines for classifying endemicity (See Table 2).

**Table 2:** Overview of usage of indicator, cut-offs, and levels for endemicity classification.

Source	Study setting	Endemicity/ Burden	Scoring method	Indicators	Classification levels						
					*Hyperendemic*	*Very high*	*High*	*Medium*	*Low*	*Undefined endemic*	*Non-endemic*
Ajalla et al. 2016 [17]	Brazil, State-level	Endemicity	Scoring single indicator Brazil method	New case detection rate	-	20–39 per 100,000 (data: 29.5 per 100,000)	-	-	-	-	-
Alencar et al. 2012a [24]	Brazil, state-level	Endemicity	Scoring multiple indicators separately Brazil method	New case detection rate	>40.00 per 100,000	-	-	-	-	-	-
				New case detection rate in children under 15 years	-	-	-	<2.5 per 100,000	-	-	-
Alencar et al. 2012b [22]	Brazil, municipality-level	Endemicity	Scoring multiple indicators separately Brazil method	New case detection rate	-	-	95 per 100,000^b^	-	-	-	-
				New case detection rate in children under 15 years	-	-	28.4 per 100,000^b^	-	-	-	-
				Rate with grade 2 disability	-	-	4.4 per 100,000^b^	-	-	-	-
Anchieta et al. 2016 [29]	Brazil, State-level	Endemicity	Scoring multiple indicators separately Brazil method	New case detection rate	51 per 100,000	-	-	-	-	-	-
				New case detection rate under 15 years	17.5 per 100,000	-	-	-	-	-	-
Barbosa et al. 2018 [30]	Brazil, Muncipality	Endemicity & burden	Scoring multiple indicators separately Brazil method	New case detection rate	>40.00 per 100,000	20.00–39.99 per 100,000	10.00–19.99 per 100,000	2.00–9.99 per 100,000	<2.00 per 100,000	-	-
				New case detection rate in children under 15 years	>10.00 per 100,000	5.00–9.99 per 100,000	2.50–4.99 per 100,000	0.50–2.49 per 100,000	<0.50 per 100,000	-	-
				New case with grade 2 disability at time of diagnosis	-	>10.00 per 100,000	5.00–9.99 per 100,000	2.00–4.99 per 100,000	0.1–1.99 per 100,000	-	-
Bernardes et al. 2017 [14]	Brazil, state-level	Endemicity	Scoring multiple indicators separately	Prevalence rate	-	-	-	4.4 per 10,000	0.73 per 10,000	-	-
				New case detection rate	-	-	-	4.76 per 100,000	-	-	-
Brito et al. 2015 [20]	Brazil, state-level	Endemicity	Scoring multiple indicators separately Brazil method	New case detection rate	-	20.00–39.99 per 100,000	-	-	-	-	-
				Proportion of leprosy cases with G2D at the time of diagnosis	-	-	>10%;	5%– 9.9%	<5%	-	-
Cunha et al. 2015 [19]	Brazil, State level	Endemicity	Scoring multiple indicators separately Brazil method	New case detection rate	>40.00 per 100,000	20.00–40 per 100,000	10.00–20 per 100,000	-	-	-	-
				New case detection rate in children under 15 years	>10.00 per 100,000	5.00–10 per 100,000	-	-	-	-	-
Da Silva et al. 2010 [26]	Brazil, town	Endemicity	Scoring single indicator Brazil method	New case detection rate	40 per 10,000	-	-	-	-	-	-
De Oliveira et al. 2012 [23]	Brazil, municipality	Endemicity	Scoring single indicator Brazil method	New case detection rate	>40 per 100,000	-	-	-	-	-	-
De Sousa et al. 2020 [31]	Brazil, district	Endemicity	Scoring single indicator Brazil method	New case detection rate	>40.00 per 100,000	20.00–39.99 per 100,000	10.00–19.99 per 100,000	2.00–9.99 per 100,000	<2.00 per 100,000	-	-
De Souza et al. 2018 [33]	Brazil, State-level	Endemicity	Scoring multiple indicators separately Brazil method	New case detection rate	>40 per 100,000	20–40 per 100,000	10–20 per 100,000	2–10 per 100,000	<2 per 100,000	-	-
				New case detection rate in children under 15 years	>10.00 per 100,000	5.00–9.99 per 100,000	2.50–4.99 per 100,000	0.50–2.49 per 100,000	<0.50 per 100,000	-	-
				Rate with grade 2 disability at time of diagnosis	> 8 per 100,000	4–8 per 100,000	2–4 per 100,000	1–2 per 100,000	< 1 per 100,000	-	-
Fontes et al. 2017 [11]	Brazil, state-level	Endemicity	Scoring single indicatorBrazil method	New case detection rate	>4.0 per 10,000	-	-	-	-	-	-
				New case detection rate in children under 15 years	-	0.5–1.0 per 10,000	-	-	-	-	-
Freitas et al. 2016 [18]	Brazil, state-level	Endemicity	Scoring multiple indicators separately Brazil method	New case detection rate	>40.00 per 100,000	20.00–39.99 per 100,000	10.00–19.99 per 100,000	2.00–9.99 per 100,000	<2.00 per 100,000	-	-
				New case detection rate in children under 15 years	>10.00 per 100,000	5.00–9.99 per 100,000	2.50–4.99 per 100,000	0.50–2.49 per 100,000	<0.50 per 100,000	-	-
				Rate of new cases with G2D at time of diagnosis	-	≥4 cases	>0–4 cases	-	0 cases	-	-
Freitas et al. 2017a [15]	Brazil, state-level	Endemicity	Scoring single indicator Brazil method	New case detection rate in children under 15 years	>10.00 per 100,000 (data: 22.7 per 100,000)	5.00–9.99 per 100,000	2.50–4.99 per 100,000	0.50–2.49 per 100,000	<0.50 per 100,000	-	-
Freitas et al. 2017b [13]	Brazil, municipality level	Endemicity	Scoring single indicator Brazil method	New case detection rate	> 40 per 100,000	-	-	-	-	-	-
Ignotti et al. 2007 [28]	Brazil, State-level	Endemicity	Scoring single indicator	Trend in proportion of new cases with a single lesion at time of diagnosis	20.3–49.1% (time series)	-	-	-	-	-	-
Imbiriba et al. 2008 [27]	Brazil, state-level	Endemicity	Scoring multiple indicators separately Brazil method	Prevalence rate	>20.0 per 10.000	10.0–20.0 per 10.000	5.0–10.0 per 10.000	1.0–5.0 per 10.000	<1.0 per 10,000	-	-
				New case detection rate	>4.0 per 10,000	2.0–4.0 per 10,000	1.0–2.0 per 10,000	0.2–1.0 per 10,000	<0.2 per 10,000	-	-
				New case detection rate in children under 15 years	1.0 per 10,000	0.5–1.0 per 10,000	0.25–0.5 per 10,000	0.05–0.25 per 10,000	<0.05 per 10,000	-	-
Moreira et al. 2014 [21]	Brazil, State level	Endemicity	Scoring multiple indicators separately Brazil method	New case detection rate	>40.00 per 100,000	20.00–39.99 per 100,000	10.00–19.99 per 100,000	2.00–9.99 per 100,000	<2.00 per 100,000	-	-
				New case detection rate in children under 15 years	>10.00 per 100,000	5.00–9.99 per 100,000	2.50–4.99 per 100,000	0.50–2.49 per 100,000	<0.50 per 100,000	-	-
				Proportion of leprosy cases with grade 2 disability at diagnosis	-	-	>10%	5–9.9%	<5%	-	-
				Prevalence rate	-	-	-	2.5 per 10,000	-	-	-
Pereira et al. 2011 [25]	Brazil, municipality	Endemicity	Scoring multiple indicators separatelyBrazil method	New case detection rate	>40.00 per 100,000	20.00–39.99 per 100,000	10.00–19.99 per 100,000	2.00–9.99 per 100,000	<2.00 per 100,000	-	-
				New case detection rate in children under 15 years	>10.00 per 100,000	-	-	-	-	-	-
Pereira et al. 2019 [32]	Brazil, municipality	Endemicity	Scoring multiple indicators separately Brazil method	New case detection rate	>40.00 per 100,000	-	-	-	-	-	-
				Proportion of children under 15 years	7.2%	-	-	-	-	-	-
				Proportion of leprosy cases with grade 2 disability	-	-	7.0%	-	-	-	-
Santos et al. 2016 [16]	Brazil, municipality-level	Endemicity	Scoring single indicator Brazil method	New case detection rate in children under 15 years	-	5.00–9.99 per 100,000	-	-	-	-	-
Aggarwal et al. 2010 [36]	India, district- & community-level	Burden	Scoring single indicator	New case detection rate (district-level)	-	-	24 per 10,000	-	-	-	-
				New case detection rate (community-level)	-	-	5 per 10,000	-	2 per 10,000	-	-
Govindharaj et al. 2019 [39]	India, district-level	Endemicity	Scoring multiple indicators separately	Prevalence rate	-	-	3.52 per 10,000	-	-	-	-
				New case detection rate	-	-	47.20 per 100,000	-	-	-	-
Kumar et al. 2007 [37]	India, district-level	Endemicity	Scoring multiple indicators separately	Prevalence rate	-	-	-	-	-	16.4 per 10,000	-
				Incidence rate	-	-	-	-	-	6.2 per 10,000 person years	-
Kumar et al. 2018 [38]	India, state-level	Endemicity	Scoring single indicator	New case detection rate	-	-	1.28 per 10,000	-	0.21 per 10,000.	-	-
Mohite et al. 2013 [35]	India, district-level	Endemicity	Scoring single indicator	Prevalence rate	-	-	-	-	-	>1 per 10,000	-
Murugaiyan et al. 2017 [34]	India, district level	Burden	Scoring single indicator	New case detection rate	-	-	>10 per 100,000	-	-	-	-
Dharmshaktu 2020 [56]	Global, country-level	Endemicity	Scoring multiple indicators separately	Prevalence rate	-	-	>1 per 10,000	-	-	-	-
				New case detection rate	-	-	>9 per 100,000	-	-	-	-
Penna et al. 2012 [54]	Global, country-level	Endemicity	Scoring multiple indicators separately	Prevalence rate	-	-	>1 per 10,000	-	-	-	-
				New case detection rate	-	-	>9 per 100,000	-	-	-	-
WER, WHO 1998 [55]	Global, country-level	Endemicity	Scoring single indicator	Prevalence rate	5–15 per 10,000	-	-	-	-	3–5 per 10,000 1–3 per 10,000	-
Basel et al. 2014 [2]	Bangladesh, district-level	Burden	Scoring single indicator	Incidence rate	-	-	3.7 per 10,000 person years at risk	-	-	-	-
Blok et al. 2018 [43]	Bangladesh, regional-level	Endemicity	Scoring single indicator	New case detection rate	-	-	25 per 100,000	5 per 100,000	1 per 100,000	-	-
Richardus et al. 2005 [50]	Bangladesh and Thailand, district- & province-level	Endemicity	Scoring single indicator	New case detection rate (average over 10 years)	-	-	50 per 100,000	-	1.5 per 100,000	-	-
Chen et al. 2007 [41]	China, province-level	Endemicity	Scoring single indicator	Prevalence rate per 100,000	-	-	-	-	-	-	<1/100,000 (referred as: dying-out)
Chen et al. 2018 [42]	China, province-level	Endemicity	Scoring single indicator	New case detection rate	-	-	1.13 per 100,000	-	-	-	-
Shen et al. 2010 [40]	China, province-level	Endemicity	Scoring single indicator	Case detection rate	-	-	-	-	-	>0.2 per 100,000	<0.2 per 100,000
Hasker et al. 2017 [44]	Comoros, island	Endemicity	Scoring single indicator	Incidence rate	-	-	7.4 per 10,000	-	-	-	-
Ortuno-Gutierrez et al. 2019 [45]	Comoros, island	Endemicity	Scoring single indicator	Incidence rate	-	-	5–10 per 10,000	-	-	-	-
Odriozola et al. 2017 [52]	Argentina, province-level	Burden	Scoring single indicator	New case detection	-	-	>100 cases	-	-	-	-
Furst et al. 2018 [46]	Cambodia, country-level	Endemicity	Scoring single indicator	New case detection rate	-	-	-	-	0.1 per 100,000	-	-
Tabah et al. 2016 [48]	Cameroon, regional-level	Endemicity & burden	Composite score with multiple indicators WHO-AFRO LBS method	Prevalence rate	-	-	>2 per 10,000	1–2 per 10,000	<1 per 10,000	-	-
				New case detection rate	-	-	>20 per 100,000	10–20 per 100,000	<10 per 100,000	-	-
				% MB among new cases	-	-	<50%	50–75%	76–100%	-	-
				% children among new cases	-	-	>20%	10–20%	<10%	-	-
				% G2D among new cases	-	-	>20%	10–20%	<10%	-	-
				% females among new cases	-	-	<40%	>60%	40–60%	-	-
				Prevalence/detection ratio	-	-	>2	1–2	<1	-	-
				Rate with grade 2 disability	-	-	>1 per 100,000	0.5–1 per 100,000	<0.5 per 100,000	-	-
Bakker et al. 2002 [51]	Indonesia, islands	Endemicity	Scoring multiple indicators separately	Prevalence rate	-	-	195 per 10,000	-	-	-	-
Dabrera et al. 2016 [47]	Sri Lanka, district-level	Endemicity	Scoring multiple indicators separately	New case detection rate	-	-	205 per 10,000	-	-	-	-
				Prevalence rate	-	-	511 per 10,000	-	-	-	-
				Child prevalence rate	-	-	1832.4 per 100,000	-	-	-	-
Aranzazu et al. 2012 [49]	Venezuela, community-level	Endemicity	Scoring multiple indicators separately	Prevalence rate	3.4 per 10,000	-	-	-	-	-	-
				Case detection rate	> 4 per 100						

^a^ Table provides an overview of scoring method and indicators used to describe endemicity and/or burden. Only indicators with cut-off values are presented. Note: 2 out of 47 articles did not provide classification cut-off: Araujo et al. 2017 [12] and Aceng et al 2019 [53]

^b^ classified as high transmission areas.

### Indicators used for measuring endemicity and burden

**Fig 2:**
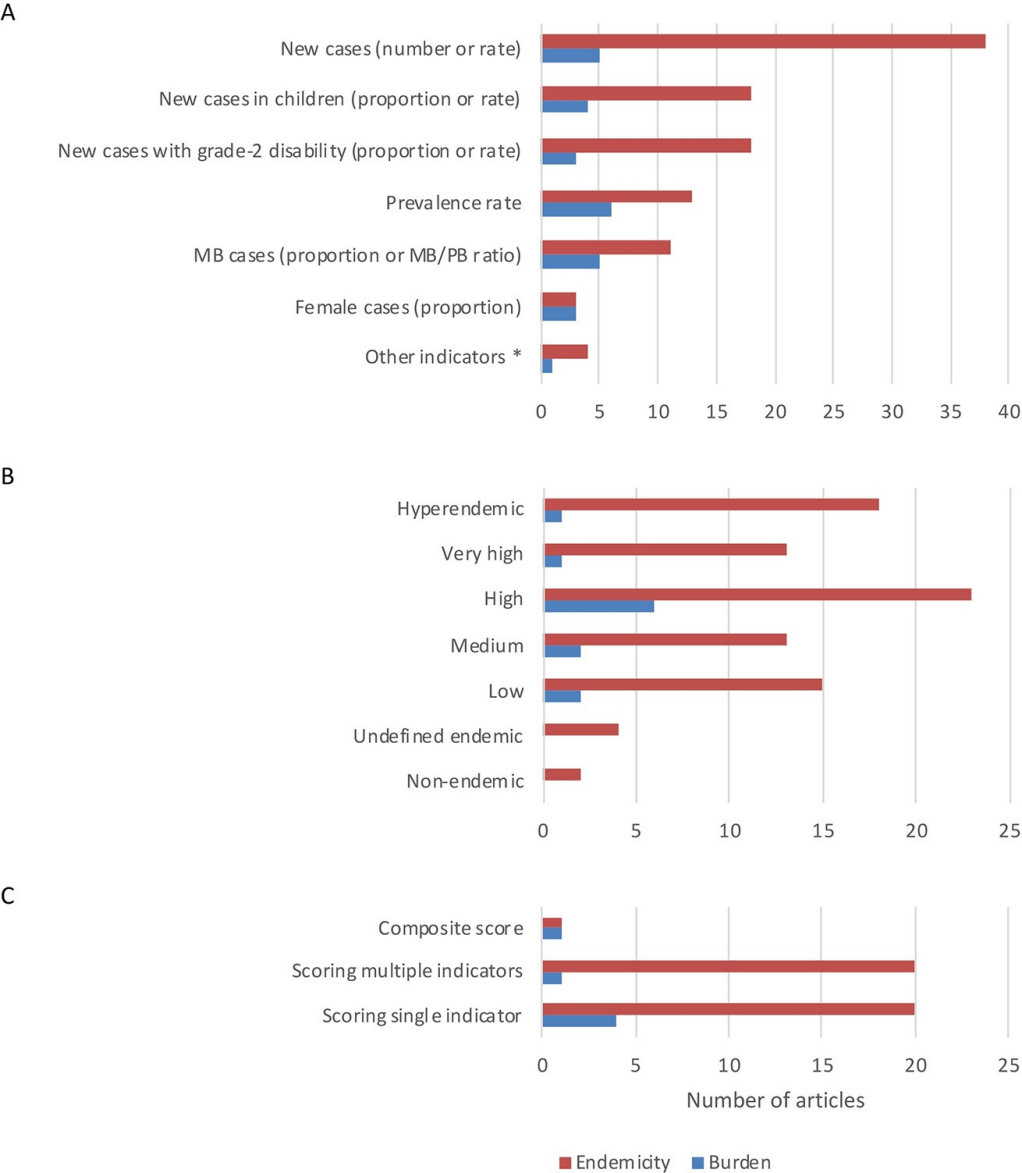
**Indicators (A), classification levels (B) and scoring methods (C) used to classify endemicity and burden of leprosy.** The bars represent the number of articles that used a particular indicator, classification level, or scoring method to classify endemicity (red) or burden (blue). An article could mention multiple indicators and classification levels. Other indicators include cure rate (2; endemicity), new cases with dimorphic clinical form (2; endemicity), trend in proportion of new cases with a single lesion at time of diagnosis (1; endemicity), and prevalence/detection ratio (1; burden).

In the literature, nine leprosy epidemiological indicators were used to determine endemicity and burden of leprosy in an area (See Fig 2A). The NCD (number and/or rate) was the most used indicator to measure endemicity (n = 38), followed by child cases (n = 18) and new cases with G2D (n = 18). To measure burden, the prevalence rate was the most used indicator (n = 6), followed by NCD (n = 5), MB cases (n = 5), child cases (n = 4) and new cases with G2D (n = 3).

The second round of the Delphi survey showed that most respondents (rank score = 4.11) ranked NCD (number and/or rate) as the most relevant indicator to classify endemicity, which was followed by NCDR in children (rank score = 2.00), and G2D among new cases (1.17) (See Table 3). To measure burden of leprosy, the top 3 ranked indicators include: prevalence of people with disabilities due to leprosy (score = 1.63), NCD (number and/or rate) (1.59), and number of reactions, neuritis, and lasting disabilities (1.13). Generally, the ranking scores of all indicators are below 2, indicating that there is no clear agreement about the choice of indicators for burden. Other indicators, such as quality of control programme and economic measures and treatment cost, but were not considered a priority for scoring endemicity and burden. A full list of all indicators can be found in S2 File.

**Table 3:** Results of Delphi survey.

Endemicity (N = 18)	Score		Burden (N = 16)	Score	
*Top indicators*	*Range 0–5* *	*% Rank Top 3*	*Top indicators*	*Range 0–5* *	*% Rank Top 3*
New case detection (number and/or rate)	4.11	88.9	Prevalence of people with disabilities due to leprosy	1.63	37.5
New cases detected among children (number and/or rate)	2.00	44.4	New case detection (number and/or rate)	1.63	31.3
Proportion of child cases among total new cases detected	1.22	27.8	Number of reactions, neuritis & lasting disabilities	1.50	31.3
Proportion of G2D cases among total new cases detected	1.17	22.2	New cases detected with G2D (number and/or rate)	1.13	18.8
New cases detected with G2D (number and/or rate)	0.89	16.7	Prevalence (number and/or rate)	1.00	18.8
New case detection trend	0.78	16.7	Proportion of G2D cases among total new cases detected	0.94	18.8
			Disability-adjusted life years	0.94	18.8
*Usage of Indicator value*	*Range*: 0–4**	*% Agree*	*Usage of Indicator value*	*Range*: *0–4*	*% Agree*
Single year value and average value of past three/five/ten years	3.3	83.3	Single year value and average value of past three or five years	3.1	75.0
Average of past three years	2.4	44.4	Average of past three or five years	3.1	87.5
Average of past five years	2.4	44.4	Single-year value	2.0	31.3
Average of past ten years	2.2	50.0			
Single-year value	1.8	38.9			
*Classification levels*	*Range*: 0–2***	*% Relevant*	*Classification levels*	*Range*: 0–2**	*% Relevant*
High	1.39	94.4	High	1.50	93.8
Low	1.39	94.4	Low	1.38	87.5
Non-endemic	1.33	83.3	No burden	1.25	75.0
Medium	1.17	77.8	Very High	1.06	68.8
Hyper	0.89	61.1	Medium	0.94	62.5
No specific level (i.e. endemic/ non-endemic)	0.72	50.0			
*Indicator cut-offs*	*Range*: 0–4**	*% Agree*	*Indicator cut-offs*	*Range*: *0–4*	*% Agree*
Usage of indicator cut-off values is essential	2.7	57.1	Usage of indicator cut-off values is essential	2.8	73.3
Indicator cut-off values should be standardized	2.9	78.6	Indicator cut-off values should be standardized	2.3	60.0
*Preferred Method of Indicator Scoring*	*Range*: 0–2***	*% Relevant*	*Preferred Method of Indicator Scoring*	*Range*: 0–2**	*% Relevant*
Score of multiple relevant indicators ^	1.24	88.2	Score of multiple relevant indicators ^	1.3	86.7
Composite score ^^	1.18	82.4	Composite score ^^	1.0	80.0
Score of a single (most relevant) indicator ^^^	0.94	70.6	Score of a single (most relevant) indicator ^^^	0.8	66.7

* based on ranking scores: rank 1 to 10; rank 1 (5 pts), 2 (4 pts), 3 (3pts), 4 (2pts), 5 (1pt), 6–10 (0 pts)

** scoring based on five categories: strongly agree (4), agree (3), neutral (2), disagree (1), strongly disagree (0)

*** scoring based on three categories: highly relevant (2); relevant (1), and not relevant (0)

^ i.e. multiple classification level: one for each indicator

^^ i.e. one overall classification level based on multiple relevant indicators

^^^ i.e. one overall classification level

### Usage of indicator values

Only single-year indicator values have been solely used to define endemicity and burden of leprosy in published literature. The Delphi survey indicated that single year value was least favoured while trends of indicator and values representing multiple years was preferred to measure endemicity and burden (See Table 3). There was no consensus about the number of years to be considered for indicator values. Moreover, trend of indicators was preferred over average of years.

### Classification levels of endemicity and burden

The classification level high is the most used in the literature for endemicity (n = 23) and burden (n = 6), followed by the levels: hyperendemic, low, medium, very high, undefined endemic, and non-endemic (See Fig 2B). Twenty-two studies use a predefined level taken from the Brazilian method, and one study followed the LBS classification. Studies without using an existing method used varying levels, including high, low, undefined endemic and non-endemic (See Table 2).

Based on the results of the second Delphi survey, the levels high, low, non-endemic and medium were considered most relevant for classifying endemicity (See Table 3). Hyperendemic and the use of no specific level were least relevant (i.e., score < 1). For classifying the burden of leprosy, high, low, no burden and very high were considered most relevant. In contrast to endemicity, the medium level was least relevant for classifying burden of leprosy.

### Scoring endemicity and burden

There was variability in the usage of indicators, levels of endemicity, and cut-off values in majority of the studies. Overall, 45 out of 47 studies classified endemicity and/or burden-based indicator cut-off values, of which 29 studies used known pre-defined cut-off values from the WHO-Afro LBS, the Brazilian method or self-defined, and 16 studies classified endemicity and/or burden based on the actual score of the indicator i.e., data-driven scores. One study used a composite score, while the other studies scored single (endemicity n = 20; burden n = 4) or multiple (endemicity n = 20; burden n = 1) indicators separately (See Fig 2C and Table 2). Two studies only mentioned the indicators used to classify endemicity/burden of the country of study but did not provide a classification cut-off. [22,52]

Respondents of the Delphi survey indicated that indicator cut-off values for scoring endemicity and burden are essential, and that these cut-off values should be standardized worldwide (See Table 3). There was no agreement about the actual proposed cut-off values of indicators to classify endemicity or burden of leprosy (See S1 File). Scoring of multiple indicators separately to classify endemicity and burden was considered most relevant, followed by using a composite score. Classification based on a single most relevant indicator was considered least relevant for both endemicity and burden. Respondents also highlighted that trend values of indicators are most useful for a scoring method, and that the total burden is generally unknown. For a burden scoring method, it was suggested that the prevalence of disability is more informative than incidence, and the discrimination and/or social exclusion and financial measures. Generally, it is important to ensure the reliability and quality of the control programme.

## Discussion

This study shows that there is great variation in the existing method for classification of endemicity and burden across countries and regions and in classification levels and indicator cut-off values. We also observed this variation among studies that do not adhere to an existing method. Using a two-round Delphi survey, we identified relevant indicators, usage of indicators, classification levels, and scoring methods to inform a standardized method to classify endemicity and burden of leprosy.

The NCDR, child case rate and G2D rate were the most important indicators to classify endemicity at national and subnational level. Prevalence did not fall into the top three of indicators both in systematic review and Delphi survey. There have been remarkable changes in the use of leprosy indicators since the 1990s. [20] During the 1990s, prevalence was the indicator of choice for monitoring the trend of leprosy as the elimination as a public health problem was defined in terms of registered prevalence. Later in the 2000s, the indicator preference shifted from prevalence rate to NCDR [54] due to the criticism that prevalence was affected by changes in case definitions or treatment duration and other operational factors. [18,20,59] Also, with the WHO target focusing on the reduction of new child cases with G2D and G2D (new case) rate, we observe a consensus of using indicators that indicate recent transmission and potential delays in diagnosis, respectively.

The top indicators for classifying burden of leprosy were new case detection, prevalence of disability, number of patients with reactions, neuritis and lasting disabilities, cases with G2D, prevalence and disability-adjusted life years. Although the systematic review and Delphi survey to an extent agreed, there was no clear consensus in the Delphi panel on which of these indicators is most relevant to reflect burden. Our findings also show a lot of overlap with indicators used to measure endemicity, which was both evident form the systematic review and survey. One explanation is the lack of full conceptual clarity of endemicity and burden. Although there was consensus that endemicity and burden are different concepts, the definitions provided by the respondents did not cover all aspects of the concept burden. Endemicity has been defined to describe frequency of disease events in which new cases of disease can arise without importation of infection from outside.[60] Burden of disease considers the total consequences of a disease in a community, including health (e.g. disabilities), social and environmental aspects and cost to an individual and society.[61]

Another possible explanation is that most burden indicators are not routinely collected. Only the number of new cases and cases with grade 2 disability at diagnosis are available in most countries. These indicators are not sufficient to reflect the full notion of burden, as leprosy is known to have a burden beyond physical impairment, including poor mental health, stigma and social participation restrictions of patients. [62] These consequences can be life-long and are likely to remain even after elimination of transmission. To understand the burden, more comprehensive data are needed including grade-1 disability, stigma, mental health, and social participation (e.g., social capital, education, and employment). These data are also necessary to improve calculations of common-used burden measures, such as Disability-Adjusted Life Years (DALYs), and cost. Currently, the disability weights for leprosy to calculate DALYs only cover physical impairment (grade-1 and 2) and do not include other consequences such as mental health. Current weights have therefore been argued to underestimate the burden of leprosy. [63,64]

Our Delphi panel favoured using the value of multiple years or trends over the use of single-year values only to measure endemicity and burden. Single year values may vary on account of random fluctuation in transmission, local migration, operational changes/factors. The moving average was also suggested as a potential solution to overcome these variations. Using trends was considered to be more informative. This is especially true for monitoring changes over time, which cannot be done based on single-year figures.

Regarding classification levels of endemicity and burden, high and low were generally most used or considered most relevant. These levels were considered most relevant for operational purposes, i.e., targeting interventions. The level non-endemic or no burden were also considered useful because it may indicate that effort should be focused on surveillance rather than services. However, this level can only be reached when for a certain period (e.g., 5 years) no new cases have been detected. There is great variability in the use of cut-off values to classify endemicity and burden. For example, the range of cut-off values used for high and low endemicity based on the NCDR were set as: ≥10 to >100 per 100,000 population for a high level, and <1 to <20 per 100,000 for a low level (See Table 2 and S1 File). This is partly influenced by the setting context, as the rates may vary greatly across countries and regions. A certain rate may be considered high in one country or region but low in another, making interpretation of cut-off values more complex. There is clearly room to standardize cut-off values for relevant indicators, which may be addressed by future research.

Nevertheless, respondents to our survey indicated to have a preference for a standardized approach worldwide, since this would support monitoring and policy decision-making. The discrepancies between countries and regions are mainly due to the population size. For example, small populations may lead to high rates, while the absolute number of cases might be low. Therefore, a uniform approach should also consider meaningful population sizes and absolute number of new cases.

There are currently two existing scoring methods for classification of endemicity and burden (WHO-AFRO LBS and the Brazilian method). One of these two proposes a composite score, while the other method suggest scoring of multiple indicators independently. Scoring multiple indicators were also considered most relevant from the Delphi study. A single indicator is not sufficient to reflect the overall endemicity and burden unless it is a composite score. A composite score was considered relevant only if data for all indicators needed for composite scoring are available from routine data collection. Another consideration is the practical utility as a composite score is more complex. The LBS composite score has been only been used once to date, which may be due to these reasons. [48] An example of a composite indicator currently part of the WHO Road map 2021–2030 is ‘the number of people requiring interventions for NTDs’. To calculate this for a disease like leprosy, data would need to include people requiring preventive chemotherapy, leprosy medicines, treatment of reactions and nerve damage, surgical treatment, and interventions to improve mental wellbeing, social participation, and inclusion. While this presents a major challenge, it shows that the discussion on measuring endemicity and burden more relevant than ever.

To the best of our knowledge, this was the first systematic review and Delphi survey that focused on measuring and classifying endemicity and burden of leprosy. The literature search was performed in seven databases, and a fair amount of experts representing most important leprosy endemic countries participated in the Delphi survey. However, this study has limitations that may affect the generalizability of our findings. First, the systematic review consist predominantly of studies from Brazil. The main reason for this is that Brazil is the only country that has a formal published scoring method for endemicity and burden. Second, as treatment outcomes are not always referred as endemicity and/or burden, we might have missed some relevant publications in our literature search, especially regarding indicators. Another limitation is the decline in response rate from 65% in the first to 45% in the second round of the Delphi survey, which may infringe the generalizability of the ranking agreement in the second round. However, the results of the survey were validated by triangulation of the findings from the systematic review which served as a complementing research approach.

## Conclusion

There is great variation in the existing method for measuring endemicity and burden across countries and regions. Creating a standardized and uniform scoring framework for endemicity and burden of leprosy is important to allow effective communication, planning and monitoring of intervention strategies, as well as comparability across countries and regions. This study highlights key findings around relevant indicators, classification levels, and scoring methods, which could guide the development of a (new) standardized uniform approach to measure and classify leprosy endemicity and burden at (sub)national level.

## Supporting information

S1 Table Search strategy. (PDF)

S1 File Delphi survey round 1. (PDF)

S2 File Delphi survey round 2. (PDF)
