# Supplementary material for: Measuring endemicity and burden of leprosy across countries and regions: A systematic review and Delphi survey
Source: PLoS Negl Trop Dis. 2021 Sep 20;15(9):e0009769. doi: 10.1371/journal.pntd.0009769 (PMC8483296; doi:10.1371/journal.pntd.0009769)
Supplement: S1 Table — (PDF) [file pntd.0009769.s001.pdf]

S1 Table Search strategy

Database	Search terms
Embase	('leprosy'/exp OR 'leprosy epidemiology'/exp OR 'leprosy control'/de OR 'Mycobacterium leprae'/de OR 'lepromin'/de OR (hansen* OR lepra* OR lepro*):ab,ti) NOT ('endemic disease'/exp OR (endemi* OR hyperendemi*):ab,ti) AND ('disease burden'/exp OR ('burden of* OR disease'):ab,ti) AND [english]/lim
Medline Ovid	(exp Leprosy/ OR Mycobacterium leprae/ OR Lepromin/ OR (hansen* OR lepra* OR lepro*).ab,ti.) AND (Endemic Diseases/ OR (endemi* OR hyperendemi*).ab,ti.) AND (disease burden/ OR (burden of* OR disease*).ab,ti.) AND english.la.
Web of science	TS=(((hansen* OR lepra* OR lepro*)) AND ((endemi* OR hyperendemi*)) AND ((burden* OR disease*)))
Cochrane CENTRAL	((hansen* OR lepra* OR lepro*):ab,ti) AND ((endemi* OR hyperendemi*):ab,ti) AND ((burden* OR disease*):ab,ti)
Lilacs	(hansen* OR lepra* OR lepro*) AND (endemi* OR hyperendemi*)
Scielo	(hansen* OR lepra* OR lepro*) AND (endemi* OR hyperendemi*)
Google scholar	hansen|lepra|leprosyendemicity|hyperendemicity|endemic|hyperendemic
