# Supplementary material for: Measuring endemicity and burden of leprosy across countries and regions: A systematic review and Delphi survey
Source: PLoS Negl Trop Dis. 2021 Sep 20;15(9):e0009769. doi: 10.1371/journal.pntd.0009769 (PMC8483296; doi:10.1371/journal.pntd.0009769)
Supplement: S1 File — (PDF) [file pntd.0009769.s002.pdf]

Delphi Survey Round 1

General information

1. Occupation (N=26)

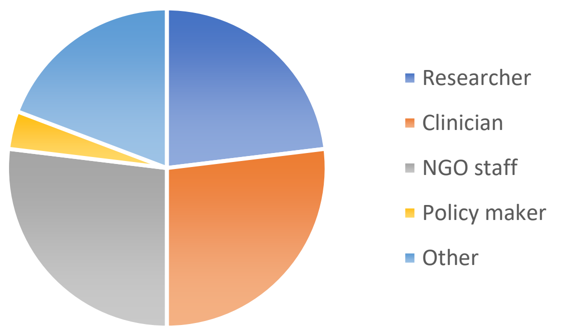

2. Country of work (N=26)

International (7)	India (6)	Nepal (3)	Sri Lanka (1)
Bangladesh (3)	Indonesia (4)	Nigeria (2)	Tanzania (3)
Brazil (5)	Ivory Coast (1)	Pakistan (1)	Thailand (1)
Cameroon (1)	Kiribati (1)	Paraguay (1)	Togo (1)
Dem. Rep. of Congo (1)	Laos (1)	Philippines (1)	Uganda (2)
Ethiopia (3)	Liberia (1)	Samoa (1)	
Gabon (1)	Mozambique (3)	Sierra Leone (1)	
Ghana (1)	Myanmar (1)	Solomon Island (1)	

Note: Number of times a country was cited is provided between brackets

3. Years of experience in the field of leprosy (N=26)

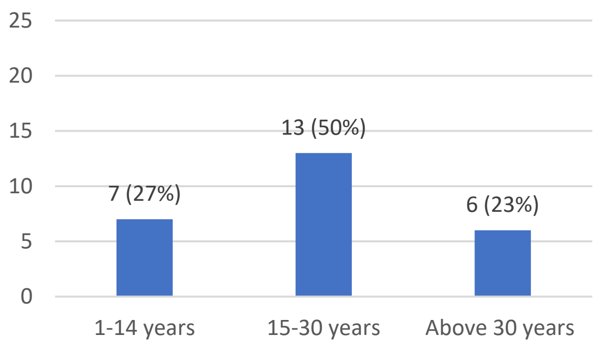

Endemicity and Burden of Leprosy

4. Do you think endemicity of leprosy and burden of leprosy are interchangeable terms/concepts? (N=26)

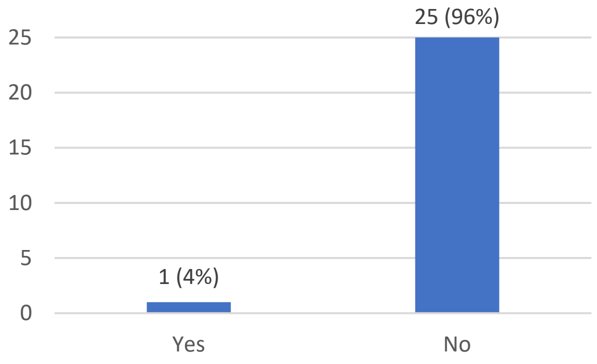

5. What does endemicity of leprosy mean to you? (N=26)

Summary of answers:

- New cases / New case detection rate (NCDR) at national and subnational level
- NCDR in children
- Prevalence at national and subnational level
- Sub-clinical infection
- Intensity of the occurrence (e.g. low and high endemic)
- Sustained transmission of leprosy to susceptible population / local transmission of M. leprae
- Number of people with undetected leprosy in a defined area
- Burden of untreated disease
- Number of cases and in need of MDT

6. What does burden of leprosy mean to you? (N=26)

Summary of answers:

- Incidence, prevalence and prevalence of disability
- Societal discrimination, psychological and social stigma and medical burden
- Cost of management, treatment, and diagnosis.
- Disability and societal consequences. Untreated and under-treated persons
- Burden of disease (morbidity, mortality)
- Quality of life: QALYS, DALYS
- Number of people diagnosed on MDT, number of smeared positive cases and relapsed cases
  Economic cost and financial loss
- Societal, individual, family and financial impact
- Direct and indirect consequences of leprosy to the affected people

7. Is there a need for additional terms/concepts besides these two? (N=26)

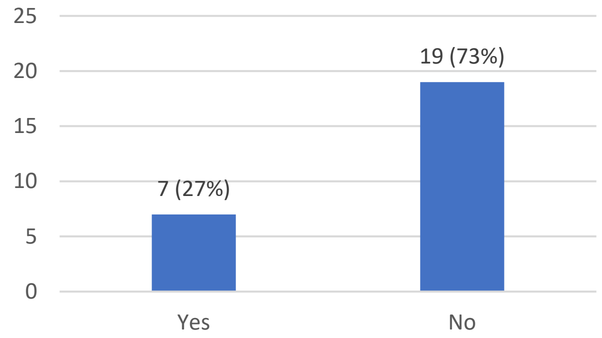

If yes, please specify and define which additional term(s)/concept(s) you would suggest (N=7)

**Term/concept**	**Description**
Societal and personal economic burden	Lifetime costs to individual and societal loss of output from affected people
Current case load	People that are in need of MDT and medical Rx
Cumulative prevalence of leprosy-related disability	Number of people living with permanent disability due to leprosy (G1D and G2D)
Cost of leprosy	-
Active case finding (ACF)	-
Quality of leprosy services	Identification and evaluation of the strength and weaknesses of the leprosy services, including social and rehabilitation services
Burden specified by a number of indicators	Number of people living with disabilities and disability related care, and social exclusion
Incidence	A measure of new cases in a segment of time
Hidden prevalence	Existing but not known cases

Measuring Endemicity and Burden of Leprosy

8. At sub-national levels in your area(s) of work, which indicator(s) is/are currently being used to define endemicity of leprosy? (N=26)

Indicator	N
Number of new cases (NCD)	12
New case detection rate (NCDR)	19
Prevalence	13
Children cases (proportion / NCDR)	11
Grade 2 disability (proportion / rate per million)	7
Female cases	4
MB cases (proportion)	4
MB cases in children	1
Patients on treatment	1
Patients with reaction	1
Number of relapse cases	1
I don't know	1

9. Do you believe that the applied indicator(s) suffice to assess endemicity of leprosy?

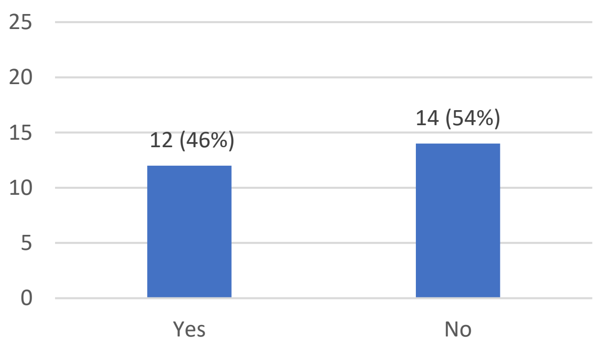

10. At sub-national levels in your area(s) of work, which indicator(s) is/are currently being used to define burden of leprosy? (N=26)

Indicator	N
Grade 2 disability (proportion / rate per million)	15
Grade 1 disability	4
Prevalence	9
New case detection rate (NCDR)	8
Cases on steroids or other disease modifying agents	1
Cases requiring surgical treatment	3
Number of new cases (NCD)	5
Children cases (proportion / NCDR)	3
Patients/Cases on treatment	4
Disability/deformity assessment after MDT completion	3
Female cases	1
MB cases (proportion)	2
Patients with reaction	2
Number of relapse cases	1
Social and financial cost	3
Prevalence of disability / cumulative disability	2
I don't know	1

11. Do you believe that the applied indicator(s) suffice to assess burden of leprosy? (N=26)

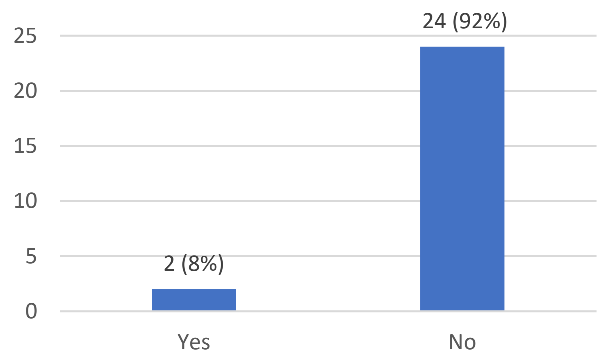

Indicators of Leprosy

12. Which of the following leprosy indicators do you consider relevant to classify endemicity of leprosy and/or burden of leprosy at sub-national level? (N=26)

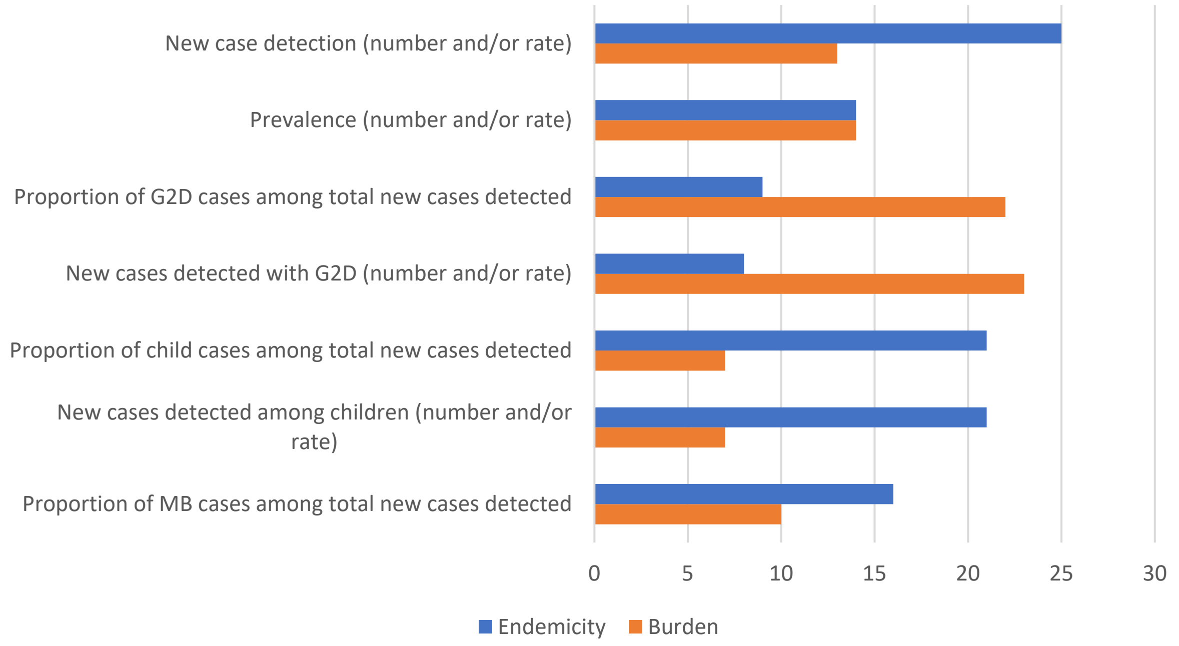

13. Can you think of any other indicator(s) that would be relevant to score endemicity of leprosy and/or burden of leprosy? These may also be indicators to assess quality of programs. (N=26)

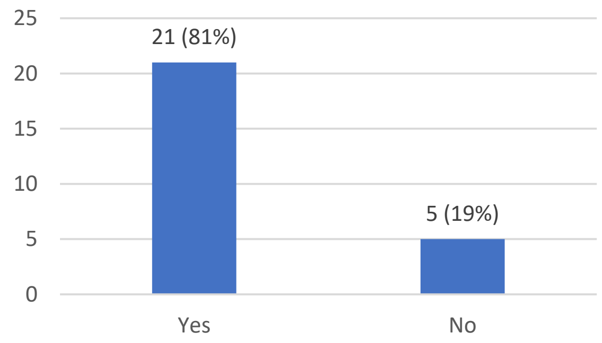

If yes, please specify which other indicator(s) you deem relevant and indicate whether the proposed indicator(s) is/are relevant to endemicity of leprosy and/or burden of leprosy:

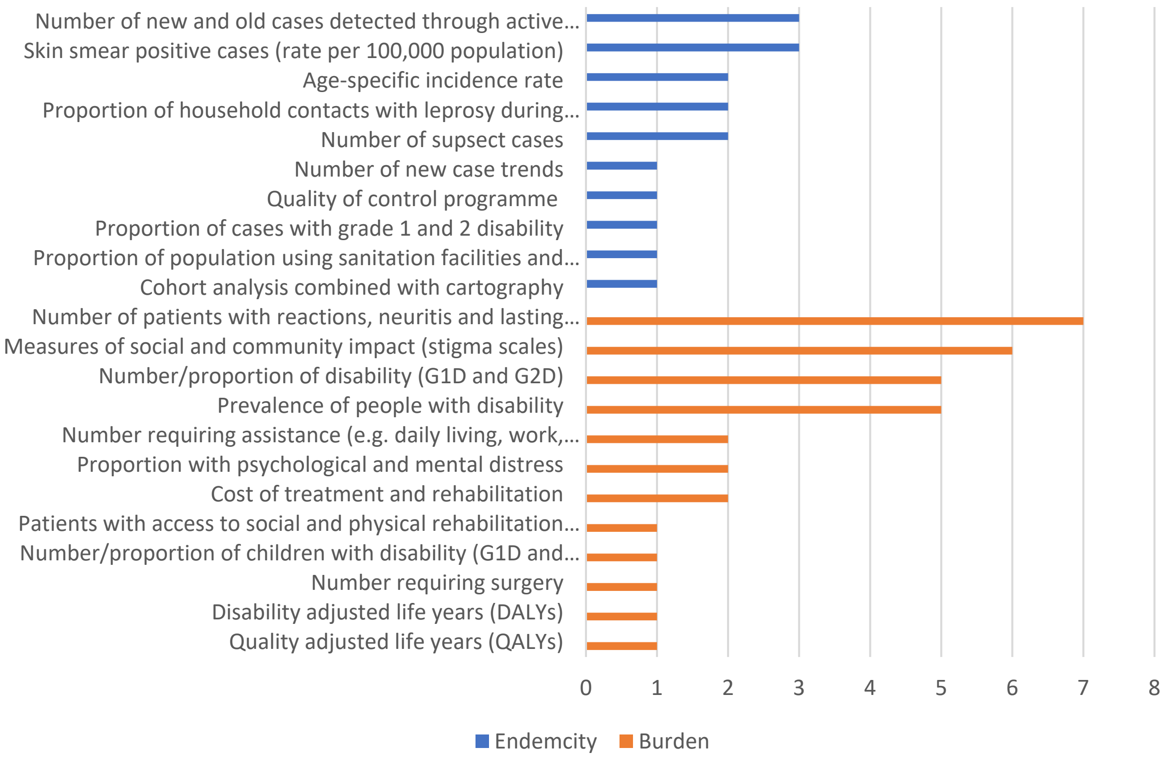

Usage of Leprosy Indicators

14. Endemicity of Leprosy: Should we use the indicator value of a single year, take the average of multiple years or use an alternative method? (N=26)

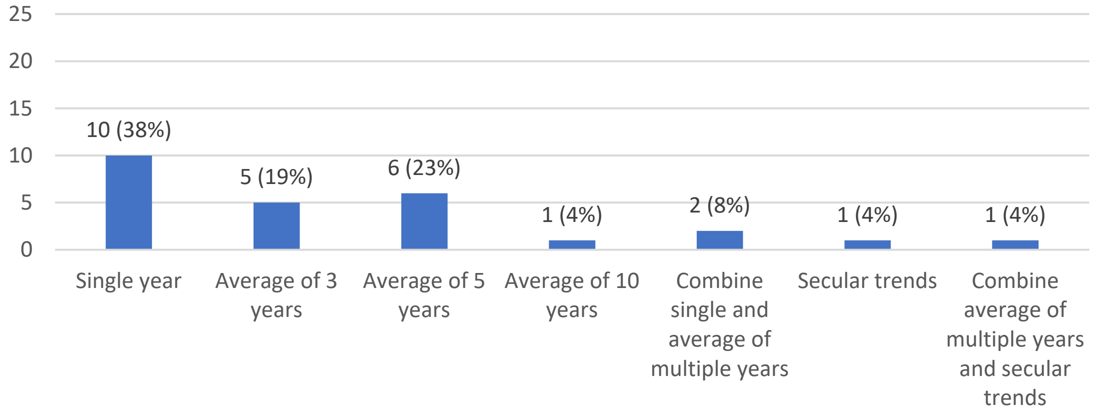

15. Burden of Leprosy: Should we use the indicator value of a single year, take the average of multiple years or use an alternative method? (N=26)

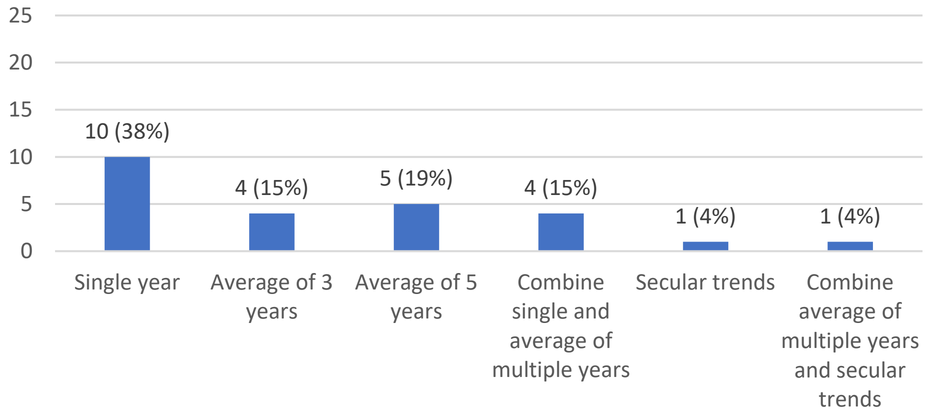

Levels of Endemicity and Burden of Leprosy

16. Which of the following levels are relevant to categorize **endemicity of leprosy** at sub-national level? *More than one answer allowed* (N=26)

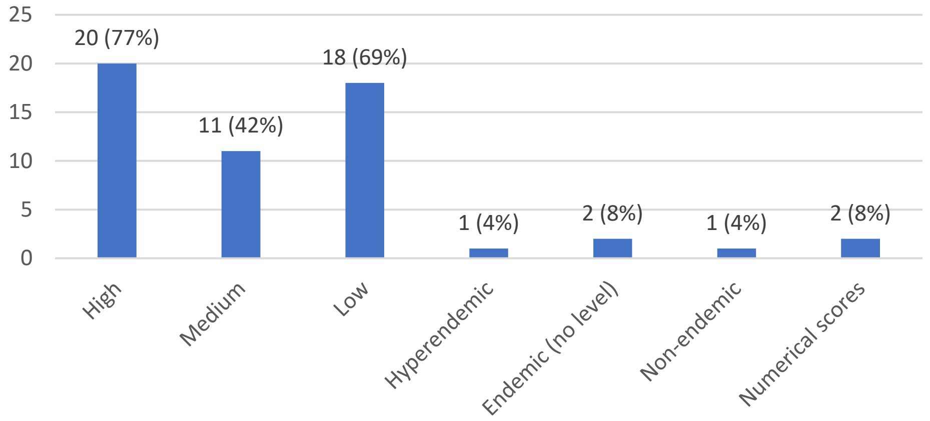

17. Which of the following levels are relevant to categorize **burden of leprosy** at sub-national level? *More than one answer allowed.* (N=26)

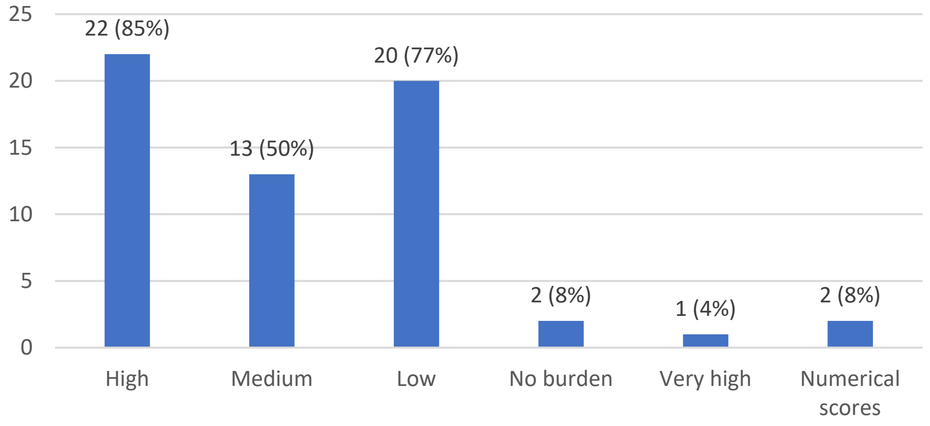

Classification of Endemicity and Burden of Leprosy

Only for previously selected/suggested indicators and level

18. Please indicate per selected indicator what you consider to be an appropriate threshold or cut-off value to identify an area for each previously selected / suggested level of **endemicity of leprosy**. (N=24)

Indicator	High	N	Medium	N	Low	N
New case detection (number and/or rate)	> 20 per 100,000 population	2	-		< 20 per 100,000 population	1
	> 10 per 100,000 population	7	1-10 per 100,000 population	5	< 1 per 100,000 population	5
	-		10 per 100,000 population	2	< 10 per 100,000 population	5
	> 10 per 10,000 population	2	1-10 per 10,000 population	1	-	
	> 1,000 new cases	1	-		< 1,000 new cases	1
	> 100,000 new cases	1	> 50,000 new cases	1	10,000 new cases	1
Prevalence (number and/or rate)	> 1 per 10,000 population	5	0.1-1 per 10,000 population	2	< 0.1 per 10,000 population	3
	> 5 per 10,000 population	2	2-5 per 10,000 population	1	2 per 100,000 population	1
	-		1-5 per 10,000 population	1	< 1 per 10,000 population	4
	> 5 per 100,000 population	1	-		-	
	> 50,000 under treatment	1	10,000-50,000 under treatment	1	< 10,000 under treatment	1
Proportion of G2D cases among total new cases detected	> 3%	1	-		< 3%	1
	>10%	4	5-10%	2	< 5%	3
	-		2-10%	1	< 2%	1
	>5%	2	-		1%	1
New cases detected with G2D (number and/or rate)	1 per 100,000 population	1	-		0.1 per 100,000 population	1
	< 1 per 1,000,000 population	1	-		-	
Proportion of child cases among total new cases detected	> 20%	2	10-20%	1	< 10%	1
	-		5-20%	1	< 5%	6
	> 10%	5	5-10%	3	-	
	-		2-10%	3	< 2%	1
	> 5%	6	1-5%	1	< 1%	4
	> 8.9% (global average)	1	-		-	
New cases detected among children (number and/or rate)	> 5 per 100,000 child population	1	2-5 per 100,000 child population	1	< 2 per 100,000 child population	1
	> 2 per 100,000 child population	1	2 per 100,000 child population	1		
	> 5 per 10,000 child population	1	1-5 per 10,000 child population	1	< 1 per 10,000 child population	1
	> 1 per 100,000 child population	2	1 per 100,000 child population	1	< 1 per 100,000 child population	2
	-		-		< 2 per 10,000 child population	1
	-		-		0.1 per 100,000 child population	1
	3 per 100,000 child population	1	-		-	
	> 5 new child cases	1	-		-	
Proportion of MB cases among total new cases detected	> 50%	3	50%	1	< 50%	1
	-		30-50%	1	< 30%	2
	> 80%	3	30-80%	1	-	
	-		20-80%	1	< 20%	2
	> 75%	2	25-75%	1	< 25%	3

	> 25%	1	-		-	
	60%	1	-		10%	1
Skin smear positive cases (proportioin or rate per 100,000 population)	> 1 per 100,000 population	1	-		< 1 per 100,000 population	1
	> 10%	2	5-10%	2	< 5%	2
Quality of control programme	High quality: active programme with contact management and preventive interventions in place	1	active programme, but with only MDT provision	1	no or hardly any leprosy control activies	1
Proportion of cases with grade 1 and 2 disability	> 5%	1	-		< 5%	1
Proportion of population using sanitation facilities and follows hygiene	80%	1	20-80%	1	< 20%	1
Number of patients accquire reactions during treatment	10%	1	-		1%	1

19. Please indicate per selected indicator what you consider to be an appropriate threshold or cut-off value to identify an area for each previously selected / suggested level of **burden of leprosy**. (N=24)

**Indicator**	**High**	**N**	**Medium**	**N**	**Low**	**N**
New case detection (number and/or rate)	> 10 per 100,000 population	3	-		< 10 per 100,000 population	1
	-		1-10 per 100,000 population	1	< 1 per 100,000 population	3
	< 1 per 100,000 population	1	-		-	
	4 per 100,000 population	1	-		-	
	> 100,000 new cases	1	50,000-100,000 new cases	1	< 50,000 new cases	1
	> 1,000 new cases		-		< 1,000 new cases	1
Prevalence (number and/or rate)	> 1 per 10,000 population	5	-		< 1 per 10,000 population	3
	-		0.1-1 per 10,000 population	2	< 0.1 per 10,000 population	2
	-		< 1 per 10,000	1		
	> 50,000 cases	1	20,000-50,000 cases	1	< 20,000 cases	1
Proportion of G2D cases among total new cases detected	> 5%	4	2-5%	1	< 2%	1
	> 20%	2	5-20%	2	< 5%	6
	> 10%	2	5-10%	1	-	
	-		-		< 1%	1
New cases detected with G2D (number and/or rate)	> 0.5 per 1,000,000 population	1	-		< 0.5 per 1,000,000 population	1
	> 1 per 100,000 population	2	-		< 1 per 100,000 population	1
	> 5 per 1,000,000 population	1	1-5 per 1,000,000 population	1	< 1 per 1,000,000 population	2
	-		1 per 1,000,000 population	1	-	
Proportion of child cases among total new cases detected	> 10%	1	5-10%	1	< 5%	1
	> 5%	1	-		< 1%	1
New cases detected among children (number and/or rate)	> 1 per 100,000 child population	2	0.5-1 per 100,000 child population	1	< 0.5 per 100,000 child population	1
	-		-		< 0.1 per 100,000 child population	1
Proportion of MB cases among total new cases detected	> 50%	1	20-50%	1	< 20%	1
	> 20%	1	5-20%	1	< 5%	1

	> 75%	1	-		< 25%	1
Number of people living with leprosy related disabilities	> 50 per 100,000 population<br><br>10 per 100,000 population<br><br>> 100,000 cases	1<br><br>1	10-50 per 100,000 population<br><br>-<br><br>-	1	< 10 per 100,000 population<br><br>1 per 100,000 population<br><br>< 100,000 cases	1<br><br>1<br><br>1
Patients with access to social and physical rehabilitation services	> 75%	1	25-75%	1	> 75%	1
Patients with reactions, neuritis and lasting disbilities	10%	1	-		1%	1
Number/proportion of disability (G1D and G2D)	> 10%	1	-		< 10%	1
Number/proportion of children with disability (G1D and G2D)	> 5%	1	-		< 5%	1

Scoring Endemicity and Burden of Leprosy

20. Do you consider a composite score, such as in the example, to be relevant for scoring endemicity of leprosy and burden of leprosy? (N=24)

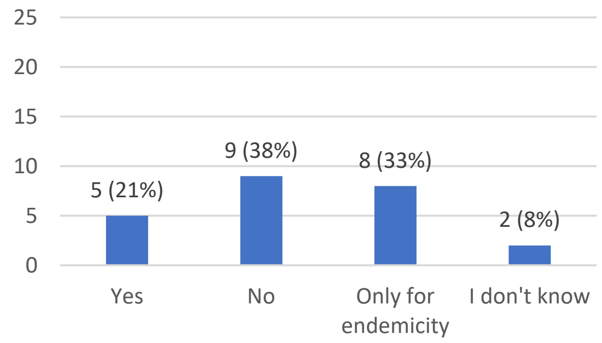

21. Do you think we need to score indicators based on threshold / cut-off values? (N=24)

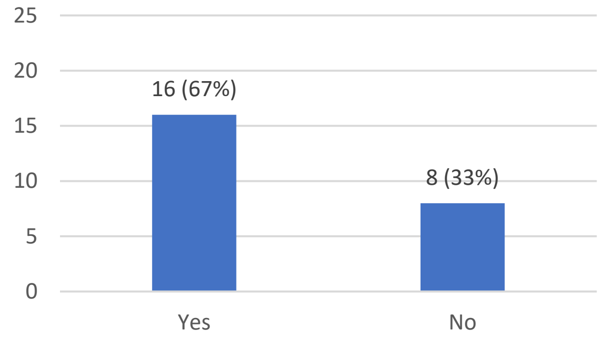

22. Do we need a separate scoring system for endemicity of leprosy and burden of leprosy or combine the two in one scoring system?

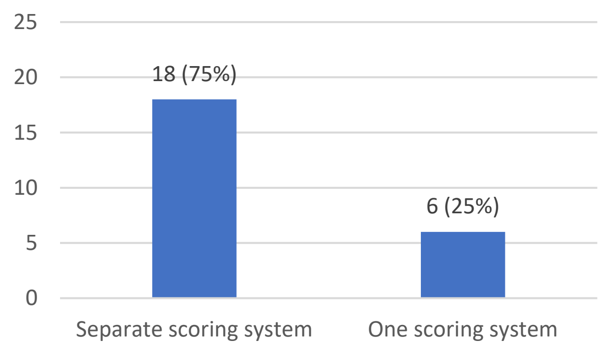

23. How would you score endemicity of leprosy? (N=18)

Summary of answers:

- Use composite score
- Use trends value of indicators (not only single years)
- Score and ranking based on data driven methods. Scores weighted by the country GDP, as disease in low-income countries places a disproportionate burden on health services.
- Score top-3 indicators: 1, 2 and 3 for low, middle and high, respectively.
- Highest priority: Number and rate of new cases detection, rate of new child cases, G2D cases
- Suggested ranking: new case detection rate, smear positive rate in population, proportion of MB cases, proportion of new cases among household contacts of a patient, rate of new child cases, proportion of children among new cases, prevalence rate
- Indictors to ensure reliability and quality of the programme: e.g. percentage of contacts screened through active case finding, percentage of suspects shown up at clinic, percentage new passive cases, percentage new cases through active case finding reported at subnational level

24. How would you score burden of leprosy? (N=17)

Summary of answers:

- Use composite score: top-5 indicators can be put into an overall score.
- Use trend values of indicators
- A system taking into account the GDP or other financial measures
- Highest priority: leprosy-related impairments/disability (particularly G2D)
- Indicators on quality control programme and level of discrimination/social exclusion
- Suggested ranking: prevalence; proportion disabled (grade 1 or 2) at RFT; proportion disabled (grade 1 or 2) at RFT among children; rate of new child cases; 5) Rate of G2D cases at diagnosis; proportion of G2D among new cases
- Prevalence of persons with disability is more informative than incidence
- Note: total burden is generally unknown
