# Supplementary material for: Measuring endemicity and burden of leprosy across countries and regions: A systematic review and Delphi survey
Source: PLoS Negl Trop Dis. 2021 Sep 20;15(9):e0009769. doi: 10.1371/journal.pntd.0009769 (PMC8483296; doi:10.1371/journal.pntd.0009769)

Delphi Survey Round 2

Indicators of Endemicity

1. Which of the following indicators are relevant to classify endemicity of leprosy at sub-national level? *Rank them in their order of importance: 1 being the most important and onward numbers being less important; two or more indicators may be ranked the same if they are equally important. Any indicator which you may consider non-relevant could be ranked as NA.* (N=18)

**Indicator**	**Ranking**										**Rank score (0-5)**	**% Rank top-3**
	**1**	**2**	**3**	**4**	**5**	**6**	**7**	**8**	**9**	**10+**		
New case detection (number and/or rate)	11	4	1								4.1	88.9
New cases detected among children (number and/or rate)	2	3	3	2	1		1				2.0	44.4
Number of new and old cases found by survey and active screening	1										0.3	5.6
New case trend	1	1	1	1			1			2	0.8	16.7
Rate of skin smear positive cases per 100,000 population	1		1			1			1	2	0.4	11.1
Age-specific incidence rate	1		1				1	1	1	1	0.4	11.1
Suspect cases (at the clinic)	1					1			2	1	0.3	5.6
Proportion of G2D cases among total new cases detected		4	1	1		2	1	1			1.2	27.8
Proportion of child cases among total new cases detected		2	1	5	1	1					1.2	16.7
New cases detected with G2D (number and/or rate)		1	3		3		1				0.9	22.2
Proportion of household contacts with leprosy during routine monitoring		1		1	1		1	1		2	0.4	5.6
Number of new and old cases found by survey and active screening			1	2	1					2	0.4	5.6
Proportion of MB cases among total new cases detected			1	1	3	2		2		1	0.4	5.6
Prevalence (number and/or rate)					1	1				2	0.1	0.0
Quality of control program (in terms of interventions)								1		3	0.0	0.0

Usage of indicators to Determine Endemicity

Please indicate to what extent you agree with the following statements:

2. In order to determine leprosy endemicity, we should use the indicator value of a single year. (N=18)

Rank score (0-4)	1.8
% Agree	38.9

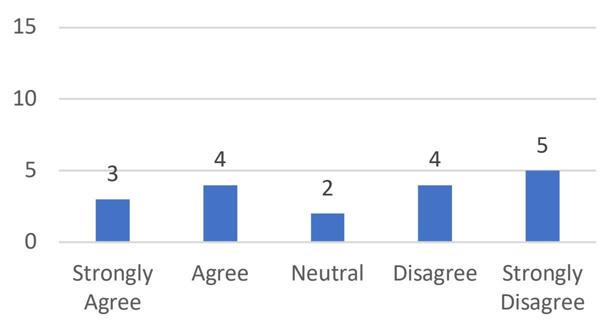

3. In order to determine leprosy endemicity, we should use the average indicator value of past three years. (N=18)

Rank score (0-4)	2.4
% Agree	44.4

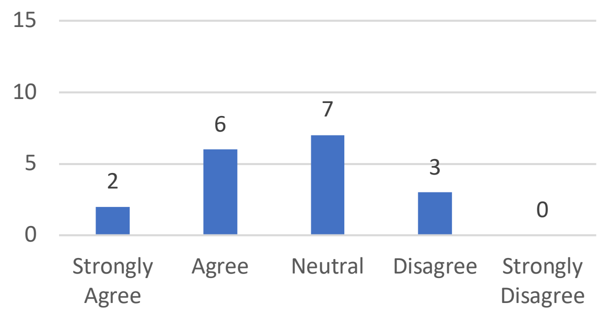

4. In order to determine leprosy endemicity, we should use the average indicator value of past five years. (N=18)

Rank score (0-4)	2.4
% Agree	44.4

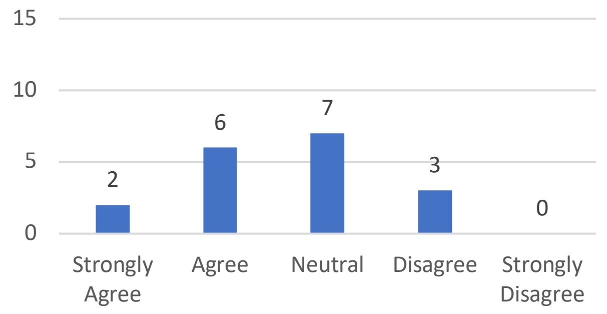

5. In order to determine leprosy endemicity, we should use the average indicator value of past ten years. (N=18)

Rank score (0-4)	2.2
% Agree	50.0

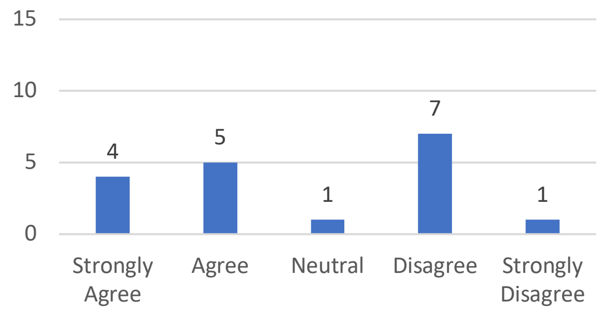

6. In order to determine leprosy endemicity, we should consider both the indicator value of a single year and the average indicator value of the past three/five/ten years. (N=18)

Rank score (0-4)	3.3
% Agree	83.3

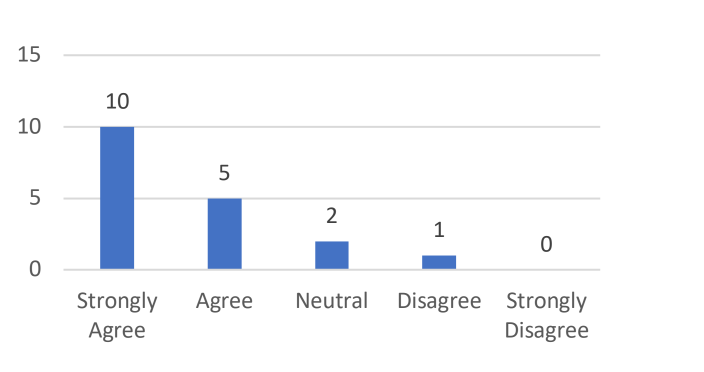

Classification Levels of Endemicity

7. Which of the following levels are relevant to categorize endemicity of leprosy at sub-national level? *Please indicate your opinion against each of the mentioned levels.* (N=15)

Hyperendemic

Rank score (0-2)	0.9
% Relevant	60.0

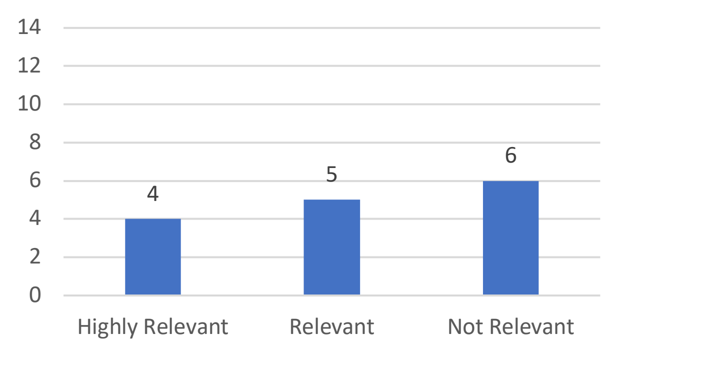

High

Rank score (0-2)	1.4
% Relevant	93.3

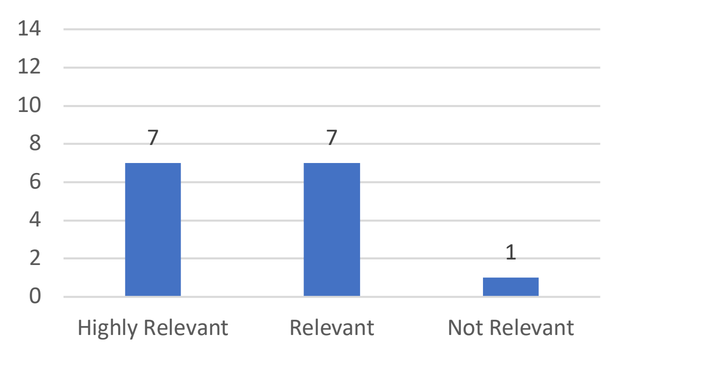

Medium

Rank score (0-2)	1.1
% Relevant	73.3

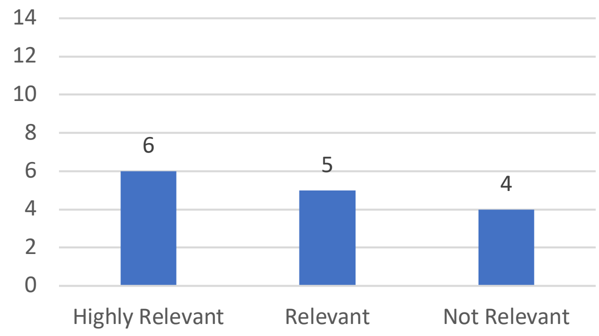

Low

Rank score (0-2)	1.4
% Relevant	93.3

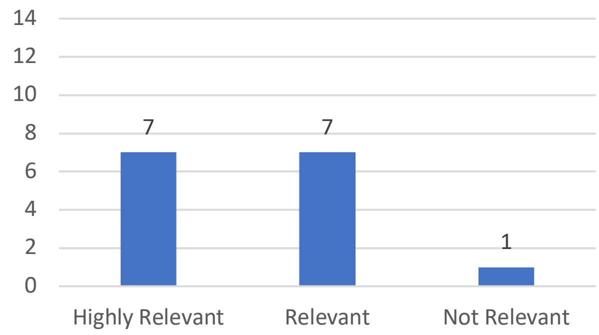

Non-endemic

Rank score (0-2)	1.3
% Relevant	80.0

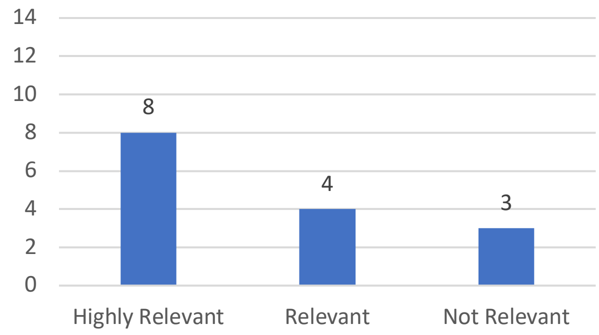

No specific level (i.e. endemic)

Rank score (0-2)	0.7
% Relevant	53.3

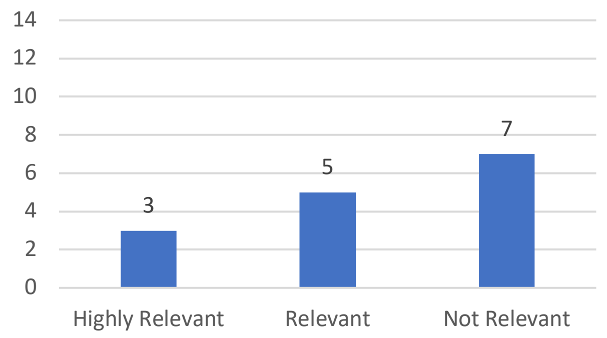

Scoring Endemicity

8. It is essential to score indicators based on threshold / cut-off values (N=14)

Rank score (0-4)	2.7
% Agree	57.1

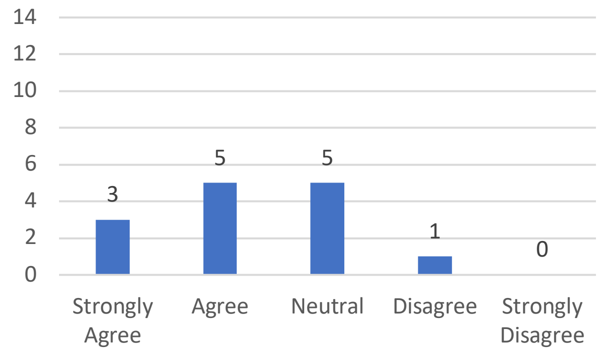

9. There should be one standard for threshold / cut-off values to score indicators at subnational-level worldwide. (N=14)

Rank score (0-4)	2.9
% Agree	78.6

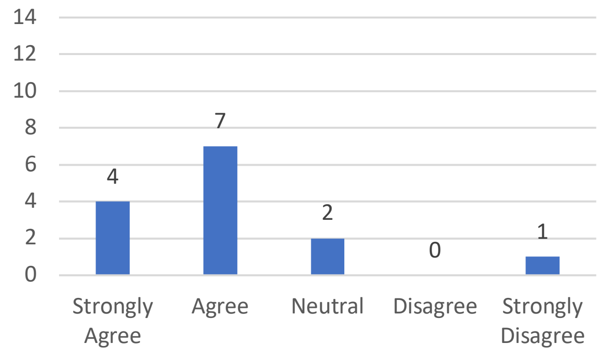

10. Which of the following scoring methods are relevant to determine endemicity of leprosy at subnational level? (N=14)

Score of a single (most relevant) indicator (i.e. one overall classification level)

Rank score (0-2)	0.9
% Relevant	71.4

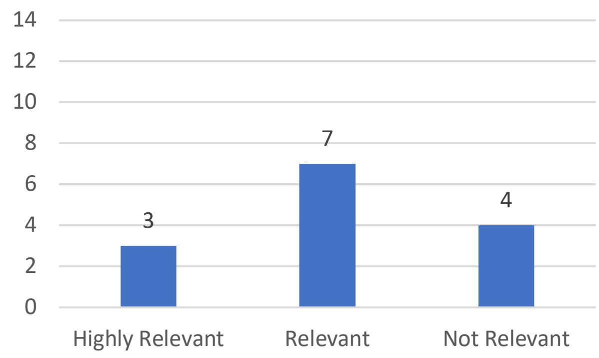

Composite score (i.e. one overall classification level based on multiple relevant indicators)

Rank score (0-2)	1.1
% Relevant	78.6

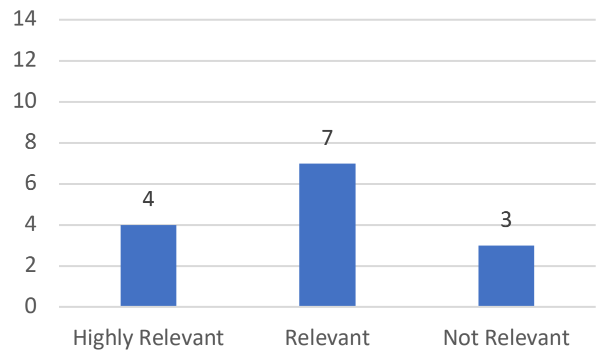

Score of multiple relevant indicators (i.e. multiple classification level: one for each indicator)

Rank score (0-2)	1.2
% Relevant	92.9

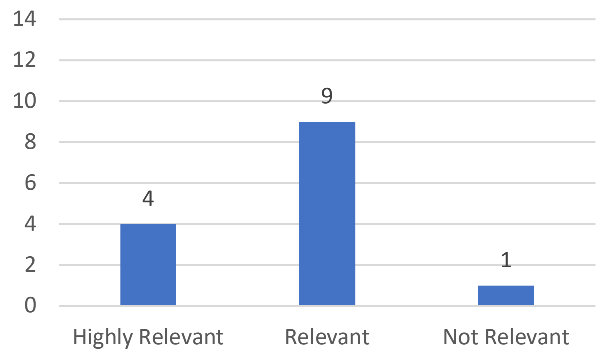

Indicators of Burden of Leprosy

11. Which of the following indicators are relevant to classify burden of leprosy at sub-national level? *Rank them in their order of importance: 1 being the most important and onward numbers being less important; two or more indicators may be ranked the same if they are equally important. Any indicator which you may consider non-relevant could be ranked as NA.* (N=16)

**Indicator**	**Ranking**										**Rank score (0-5)**	**% Rank top-3**
	**1**	**2**	**3**	**4**	**5**	**6**	**7**	**8**	**9**	**10+**		
New case detection (number and/or rate)	3	2	1							3	1.4	33.3
Disability adjusted life year (DALY)	2		1	1		2				4	0.8	16.7
Prevalence (number and/or rate)	2	1		1			1			2	0.9	16.7
Prevalence of people with disabilities due to leprosy	4		1	1	1					1	1.4	27.8
New cases detected with G2D (number and/or rate)	1	1	1	2	2				1	0	1.0	16.7
Number of reactions, neuritis & lasting disabilities	1	3	1	2			1	1	1	1	1.3	27.8
Number of children with disabilities caused by/ after treatment	1		1	1		3		1		2	0.6	11.1
Proportion of G2D cases among total new cases detected	2		1		2	1				1	0.8	16.7
Quality adjusted life year (QALY)		2				1	1		1	2	0.4	11.1
Number requiring assistance (daily living, household duties, work, etc.)		2	1		2			2	1	1	0.7	16.7
Number of disabilities caused by/ after treatment		1	2		1		2			2	0.6	16.7
New cases detected among children (number and/or rate)		2								2	0.4	11.1
Proportion of child cases among total new cases detected			1							3	0.2	5.6
Proportion of MB cases among total new cases detected			1	1		1				3	0.3	5.6
Number requiring surgery				1			2		1	5	0.1	0.0
Proportion with psychological & mental distress				1	1		1	1	2	3	0.2	0.0
Measures of social & community impact (using stigma scales)					1	1	1	2	1	2	0.1	0.0
Cost of treatment & rehabilitation						1		2		5	0.0	0.0

Usage of Indicators to Determine Burden of Leprosy

12. In order to determine leprosy burden, we should use the indicator value of a single year. (N=16)

Rank score (0-4)	2.0
% Agree	31.3

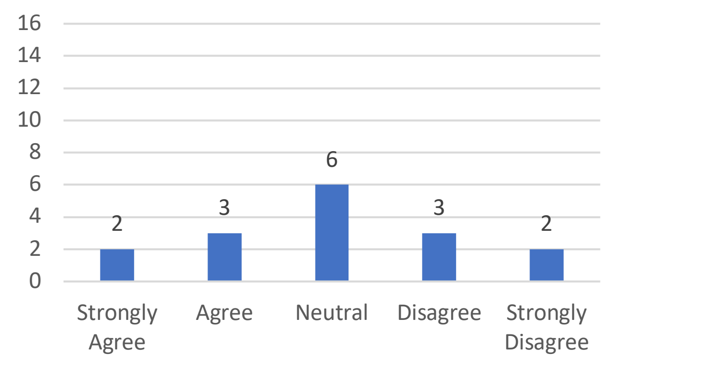

13. In order to determine leprosy burden, we should use the average indicator value of past years (e.g. thee or five years). (N=16)

Rank score (0-4)	3.1
% Agree	87.5

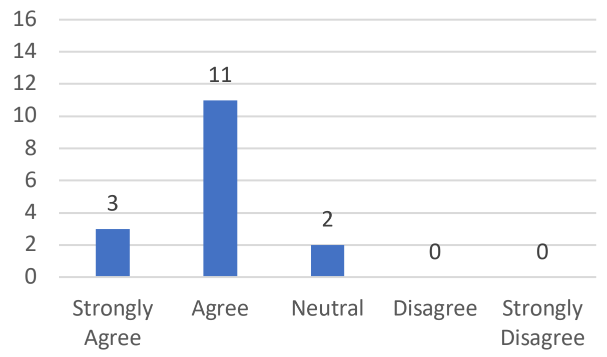

14. In order to determine leprosy burden, we should consider both the indicator value of a single year and the average indicator value of the past three or five years. Burden is determined by the highest of the two. (N=16)

Rank score (0-4)	3.1
% Agree	75.0

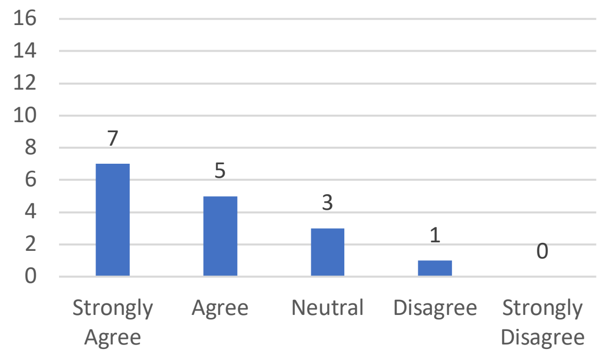

Classification Levels of Burden of Leprosy

15. Which of the following levels are relevant to categorize burden of leprosy at sub-national level? Please indicate your opinion against each of the mentioned levels. (N=16)

Very High

Rank score (0-2)	1.1
% Relevant	68.8

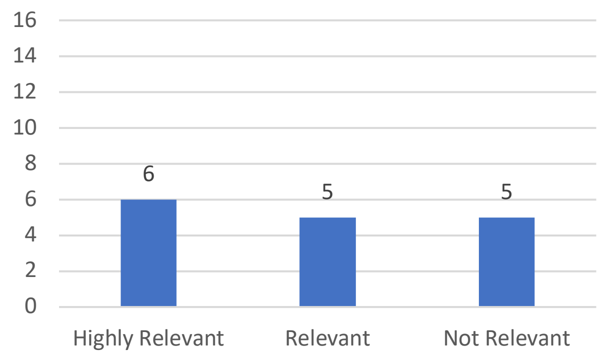

High

Rank score (0-2)	1.5
% Relevant	93.8

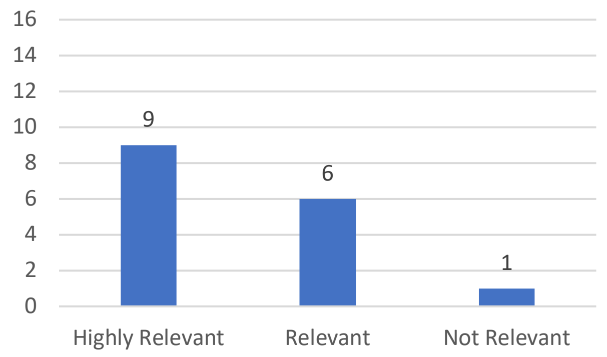

Medium

Rank score (0-2)	0.9
% Relevant	62.5

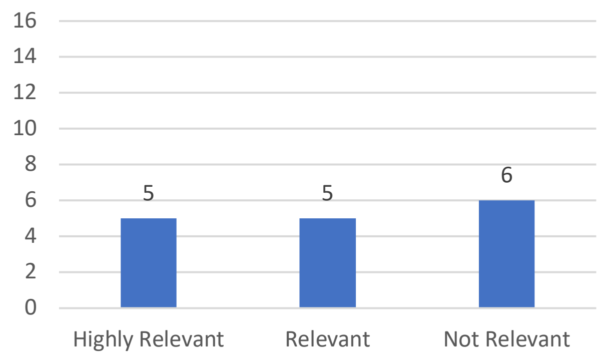

Low

Rank score (0-2)	1.4
% Relevant	87.5

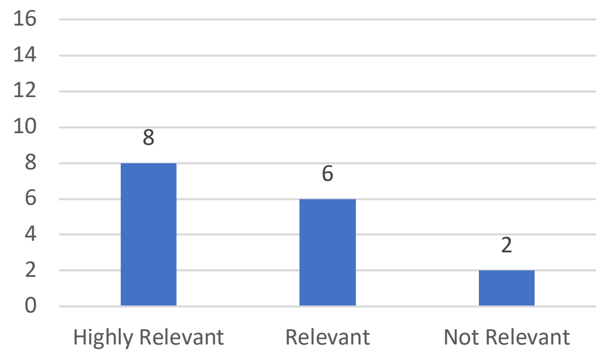

No burden

Rank score (0-2)	1.3
% Relevant	75.0

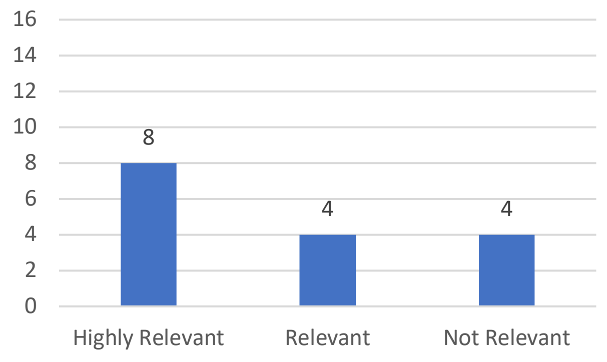

Scoring Burden of Leprosy

16. It is essential to score indicators based on threshold / cut-off values. (N=15)

Rank score (0-4)	2.8
% Agree	73.3

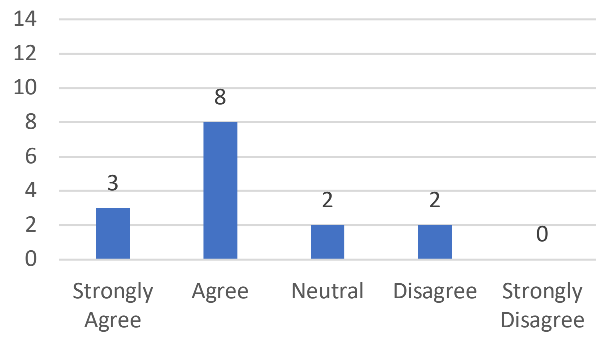

17. There should be one standard for threshold / cut-off values to score indicators at subnational-level worldwide. (N=15)

Rank score (0-4)	2.3
% Agree	60.0

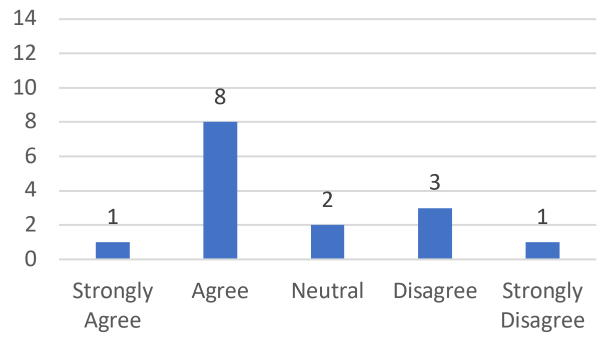

18. Which of the following scoring methods are relevant to determine burden of leprosy at subnational level? (N=15)

Score of a single (most relevant) indicator (i.e. one overall classification level)

Rank score (0-2)	0.8
% Relevant	66.7

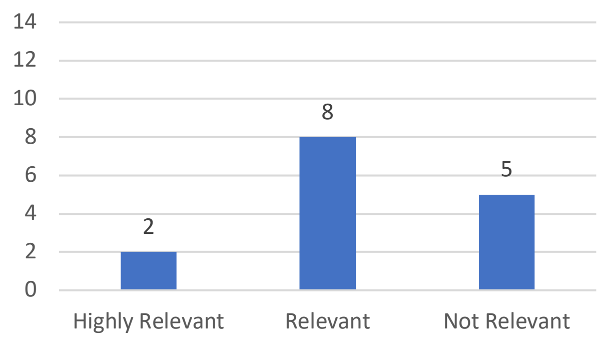

Composite score (i.e. one overall classification level based on multiple relevant indicators)

Rank score (0-2)	1.0
% Relevant	80.0

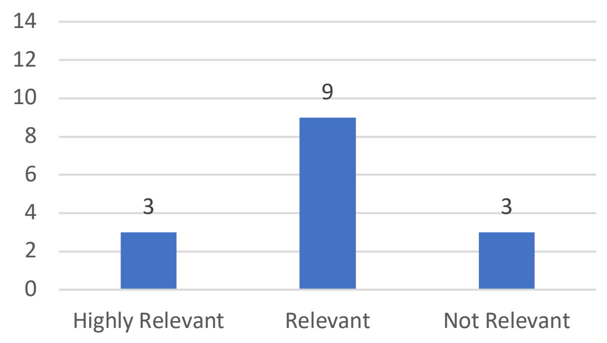

Score of multiple relevant indicators (i.e. multiple classification level: one for each indicator)

Rank score (0-2)	1.3
% Relevant	86.7

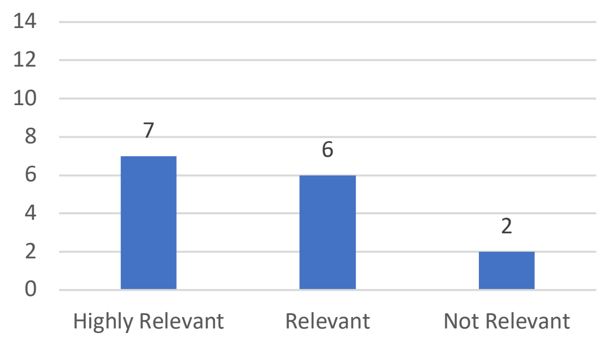

Supplement: S2 File — (PDF) [file pntd.0009769.s003.pdf]
